# Partial Loss of Genomic Imprinting Reveals Important Roles for Kcnq1 and Peg10 Imprinted Domains in Placental Development

**DOI:** 10.1371/journal.pone.0135202

**Published:** 2015-08-04

**Authors:** Erik Koppes, Katherine P. Himes, J. Richard Chaillet

**Affiliations:** 1 Magee-Womens Research Institute, Program in Integrative Molecular Biology, University of Pittsburgh School of Medicine, Pittsburgh, Pennsylvania, United States of America; 2 Magee-Womens Research Institute, Department of Obstetrics, Gynecology and Reproductive Sciences, University of Pittsburgh School of Medicine, Pittsburgh, Pennsylvania, United States of America; University of Bonn, Institut of experimental hematology and transfusion medicine, GERMANY

## Abstract

Mutations in imprinted genes or their imprint control regions (ICRs) produce changes in imprinted gene expression and distinct abnormalities in placental structure, indicating the importance of genomic imprinting to placental development. We have recently shown that a very broad spectrum of placental abnormalities associated with altered imprinted gene expression occurs in the absence of the oocyte–derived DNMT1o cytosine methyltransferase, which normally maintains parent-specific imprinted methylation during preimplantation. The absence of DNMT1o partially reduces inherited imprinted methylation while retaining the genetic integrity of imprinted genes and their ICRs. Using this novel system, we undertook a broad and inclusive approach to identifying key ICRs involved in placental development by correlating loss of imprinted DNA methylation with abnormal placental phenotypes in a mid-gestation window (E12.5-E15.5). To these ends we measured DNA CpG methylation at 15 imprinted gametic differentially methylated domains (gDMDs) that overlap known ICRs using EpiTYPER-mass array technology, and linked these epigenetic measurements to histomorphological defects. Methylation of some imprinted gDMDs, most notably *Dlk1*, was nearly normal in mid-gestation DNMT1o-deficient placentas, consistent with the notion that cells having lost methylation on these DMDs do not contribute significantly to placental development. Most imprinted gDMDs however showed a wide range of methylation loss among DNMT1o-deficient placentas. Two striking associations were observed. First, loss of DNA methylation at the *Peg10* imprinted gDMD associated with decreased embryonic viability and decreased labyrinthine volume. Second, loss of methylation at the *Kcnq1* imprinted gDMD was strongly associated with trophoblast giant cell (TGC) expansion. We conclude that the *Peg10* and *Kcnq1* ICRs are key regulators of mid-gestation placental function.

## Introduction

The process of genomic imprinting establishes and maintains parental alleles in opposing epigenetic states resulting in expression of imprinted genes from just one parental allele. This monoallelic imprinted gene expression is determined by inherited parent-specific DNA methylation patterns at autosomal gametic differentially methylated domains (gDMDs) that are perpetuated in the embryo such that one parental allele is methylated and the other is unmethylated. The epigenetic information inherited on gDMDs is thought to be critical for the control of imprinted gene expression patterns because they overlap or are adjacent to imprinting control regions (ICRs), the sequences defined genetically in humans and mice as required for allele-specific expression of many linked imprinted genes [1]. There are 24 confirmed imprinted gDMDs in mouse (21 maternal and 3 paternal), most of which are conserved in humans [2]. Propagation of imprinted gDMD methylation during preimplantation development is catalyzed by a combination of somatic and oocyte-specific isoforms of the maintenance DNA methyltransferase (DNMT1s and DNMT1o) [3]. Partial disruption of genomic imprint inheritance during preimplantation, through maternal deletion of DNMT1o, permanently ablates affected gDMD methylation from embryonic and extra-embryonic lineages and directly results in biallelic expression or repression of nearby clusters of imprinted genes [4,5].

The importance of genomic imprinting to fetal growth and development is evident when monoallelic expression is altered. The overgrowth syndrome Beckwith Wiedemann (BWS: OMIM 130650) and the growth restriction syndrome Silver-Russell (SRS: OMIM 180860) are caused by aberrant imprinted gene dosage at chromosome 11p15.5 [6–10]. Causes include uniparental disomies (UPD), reciprocal translocations, imprinted gene mutations or epigenetic mutations resulting in two alleles with the same imprinted status. Many of the imprinted genes of the *Kcnq1* and *H19* clusters that are associated with BWS and SRS are expressed and function in the placenta [11,12], and it is possible that BWS and SRS phenotypes are influenced by loss of imprinting within the placenta [13]. For example, the fetal lethality associated with deletion of the *Ascl2* gene in the mouse *Kcnq1* cluster is due to minimal placenta labyrinth development and accompanying accumulation of trophoblast giant cells (TGCs) at E10.5 [14]. Deletion of either the *Phlda2* or *Cdkn1c* genes, which also reside in the *Kcnq1* cluster, results in placental overgrowth [15,16] and transgenic over-expression of either *Phlda2* or *Cdkn1c* results in poor growth of the placenta [17–19]. Placenta growth and development is also dependent on *Igf2*, a component of the *H19* imprinting cluster; deletion of *Igf2* results in placental and fetal growth restriction and overexpression of *Igf2* produces a large placenta and accompanying fetus [20–22]. In addition, deletion of other imprinted genes not within the *Kcnq1* or *H19* clusters exhibit abnormal placental phenotypes. For example, deletion of *Grb10*, *Igf2r*, or *Mest* alters placental growth and deletion of either *Peg10* or *Rtl1* disrupts labyrinth development [23–27].

The *Dnmt1* ^*Δ1o*^ maternal effect mouse model of loss of genomic imprinting is a unique system to probe the essential role of imprinted gDMD methylation in placental development. Embryos derived from homozygous *Dnmt1* ^*Δ1o/Δ1o*^ dams lacking the oocyte isoform of DNA-methyltransferase-1 (DNMT1o) are comprised of an epigenetic mosaic of cells with partial and highly variable loss of imprinted DNA methylation [3–5]. Unlike mouse models of *Dnmt1* inactivating mutations, which exhibit severe reduction in global DNA methylation and arrest development at embryonic day 8.5 (E8.5) [28,29], progeny of *Dnmt1* ^*Δ1o/Δ1o*^ dams frequently survive through mid-gestation, albeit with profound embryonic and placental defects [30,31]. Early *Dnmt1* ^*Δ1o*^ maternal effect placental abnormalities are worse in female conceptuses due to defective X-chromosome inactivation [32].

In principle, the wide spectrum of phenotypes and highly variable patterns of gDMD methylation in progeny of *Dnmt1* ^*Δ1o/Δ1o*^ dams are associated. In clinical studies the application of quantitative imprinted gDMD methylation analysis has revealed meaningful associations between abnormal gDMD methylation and specific BWS and SRS phenotypes [33,34]. The *Dnmt1* ^*Δ1o*^ maternal effect model provides a means to define relationships between variable loss of DNA methylation at multiple gDMDs and overt placental phenotypes. This notion is supported by our previous finding that the ratio of fetal to placental weight at E17.5 is associated with changes in expression of *Ascl2* and *Mest*, presumably brought about by changes in gDMD methylation [31].

Previously we demonstrated wide-ranging placental abnormalities in DNMT1o-deficient placentas at early (E9.5) and late (E17.5) gestational times [31]. At E9.5 mutant placentas were prone to TGC accumulation and disorganized labyrinth development. Late in gestation DNMT1o-deficient placentas had greater spongiotrophoblast content and reduced labyrinth vascular surface area. In our most recent work [35] we found E17.5 DNMT1o-deficient placentas to accumulate excess lipids and have dysfunctional mitochondrial metabolism. We revealed a strong association between loss of methylation at the *Mest* gDMD and triacylglycerol levels by regression analysis. In our current study we sought to discern which genomic imprints when lost have the greatest adverse effect on placenta development and function at mid-gestational time points between E12.5 and E17.5.

## Materials and Methods

### Ethics Statement

This research was approved by the Institutional Animal Care and Use Committee of the University of Pittsburgh.

### Mouse Colony and Placenta Dissections

The *Dnmt 1* ^*Δ1o*^ mouse colony was maintained under IUPAC guidelines on a 129/Sv strain background (Taconic). Pregnant *Dnmt1* ^*Δ1o/ Δ1o*^ dams were sacrificed at 12.5, 15.5 or 17.5 days post copulation. Conceptuses were dissected to isolate the fetus, yolk sac, and placenta under a MZ12.5 dissection microscope (Leica). Placental and fetal wet weights were measured. Maternal decidua caps were removed from placental portions designated for nucleic acid and lipid extractions but not from portions for histological analysis. Each placenta was cut into halves for preservation in 4% paraformaldehyde (PFA) for histology or in RNA Later (Life Technologies) for nucleic acid extraction.

### Histology and in situ hybridization

Following fixation in 4% PFA, placental halves were suspended through a sucrose gradient up to 20% weight per volume, and then embedded in Tissue-Tek O.C.T compound (Sakura). Placental cryosections of 5μm and 10μm thickness were cut with a CM1850 cryostat (Leica) for histological analysis. Regressive hematoxylin and eosin staining was performed on a series of 5 micron meridian placental sections. A series of 10μm sections were stained by *in situ* hybridization (ISH) with Digoxigenin-11-dUTP (Roche) labeled antisense RNA probes. ISH probes of the placental marker genes *Tpbpa*, *LepR*, *Pchdh12*, *Mest*, *Prl2c2*, *Prl3b1 and Prl3d1* were in-vitro transcribed (Promega) from cDNA cloned into pBluescript, and used to identify the spongiotrophoblast (*Tpbpa*), syncytial trophoblast (*LepR*), glycogen (*Pchdh12*), fetal vascular (*Mest*) and trophoblast giant cells (*Prl2c2*, *Prl3b1 and Prl3d1*) respectively.

### Stereology and Morphometrics

All images of placental tissue sections were taken using a DMI4000B inverted microscope (Leica). Morphometric area measurements were made using the Image J (NIH) grid tool. Labyrinth and spongiotrophoblast areas were determined using random grid sampling within 2–3 central 50x or 16x fields of view of H&E stained sections for E12.5 and E15.5 placentas. Labyrinth and central volumes were calculated as the integral of area across the known distance between central H&E stained sections. Area and volume measurements were confirmed by analysis of adjacent slides stained by ISH of lineage markers. Trophoblast giant cell count measurements were from 2–3 central 10μm DAPI stained sections. The average cell count per slide was used as the reported metric. The identity of trophoblast giant cells was confirmed with ISH of adjacent sections.

### Methylation Analysis

Methylation analysis of imprinted gDMDs was carried out on all intact placentas for which non-degraded genomic DNA was recovered irrespective of fetal viability. Both DNA and RNA were purified using an AllPrep kit from Qiagen. Genomic bisulfite conversion, bisulfite converted genomic PCR, and EpiTYPER (TM—Sequenom) mass-array DNA methylation analysis was performed at the Center for Genetics and Pharmacology at the Roswell Park Cancer Institute. Pre-validated bisulfite PCR primers for imprinted gDMD genomic regions were used for the imprinted methylation analysis (S1 Table). All bisulfite amplicon sequences overlapped known primary imprinted gDMDs ([2], and references therein). Bisulfite converted PCR amplification primers for all but *H19* were chosen from a publicly available mouse imprinted panel (Sequenom). *H19* primer sequences were originally published by McGraw et al. (2013) [32]. Each EpiTYPER amplicon was validated by our internal control wild-type placenta DNA (50% imprinted gDMD methylation), *Dnmt1-*null (*Dnmt1* ^c/c^) ES cell DNA (0% imprinted gDMD methylation) and 1:2 (16.6% imprinted gDMD methylation) and 2:1 (33.3% imprinted gDMD methylation) mixtures of the two. Only amplicons that produced a linear relation between control genomic DNA expected and observed methylation fractions were selected for use in this study.

### Biostatistics and Bioinformatics

EpiTYPER absolute methylation levels were calculated as the unweighted average CpG methylation fraction across each individual imprinted gDMD amplicon. Overall imprinted gDMD methylation was determined from 12 non-redundant gDMD EpiTYPER amplicons (S1 Table) To determine if the wild-type and mutant sample methylation levels were normally distributed Kolmogorov-Smirnov, Shapiro-Wilk and Anderson-Darling tests of normality were applied to the data in SPSS (IBM) and Matlab (Mathworks). Because the mutant data were non-normally distributed we compared distributions using a Mann-Whitney U (Rank-Sum) test. Bar graphs and scatter plots of overall and individual imprinted gDMD methylation levels were originally generated with SPSS and Matlab and then adapted into Adobe Illustrator.

To display the variability in gDMD methylation intrinsic to the *Dnmt1* ^*Δ1o*^ maternal effect model we constructed heat maps. Mutant imprinted gDMD methylation levels were normalized to wild-type by dividing each sample’s imprinted gDMD absolute methylation fraction by the average wild-type methylation level for that imprinted gDMD and gestational age. The relative methylation levels were then log2 transformed and clustered using the clustergram function in Matlab. Each clustergram was adapted into a grey-scale Adobe Illustrator file.

Mean mutant and wild-type phenotypic averages were calculated. The phenotypic data was also subdivided into dead/alive and male/female subgroups to determine the influence of fetal viability and sex on placental phenotypes. Because the mutant phenotypic data were non-normally distributed the Mann-Whitney U (Rank-sum) test was used to compare the distribution of mutant and wild-type phenotypes as well as the phenotypes observed in subgroups. Phenotypic data is displayed in charts showing mean + SEM.

To associate individual placental gDMD methylation defects with particular placental phenotypic abnormalities we performed regression analyses in Matlab. Logistic regression was performed to find associations between individual imprinted gDMD methylation levels and the binary fetal viability variable. Bivariate linear regression analysis was used to associate imprinted gDMDs with the continuous phenotypic metrics for labyrinth volume, spongiotrophoblast volume, trophoblast giant cell count and fetal/placental weights. Stepwise forward linear regression modeling was performed to generate models that explain the *Dnmt1* ^*Δ1o*^ maternal effect phenotypic variation based on DNA methylation of the least number of significant gDMDs. To visually confirm strong associations (P<0.05) identified by bivariate regression we plotted placental phenotypic metrics against gDMD methylation.

## Results

### Imprinted gDMD methylation analysis

**Fig 1:**
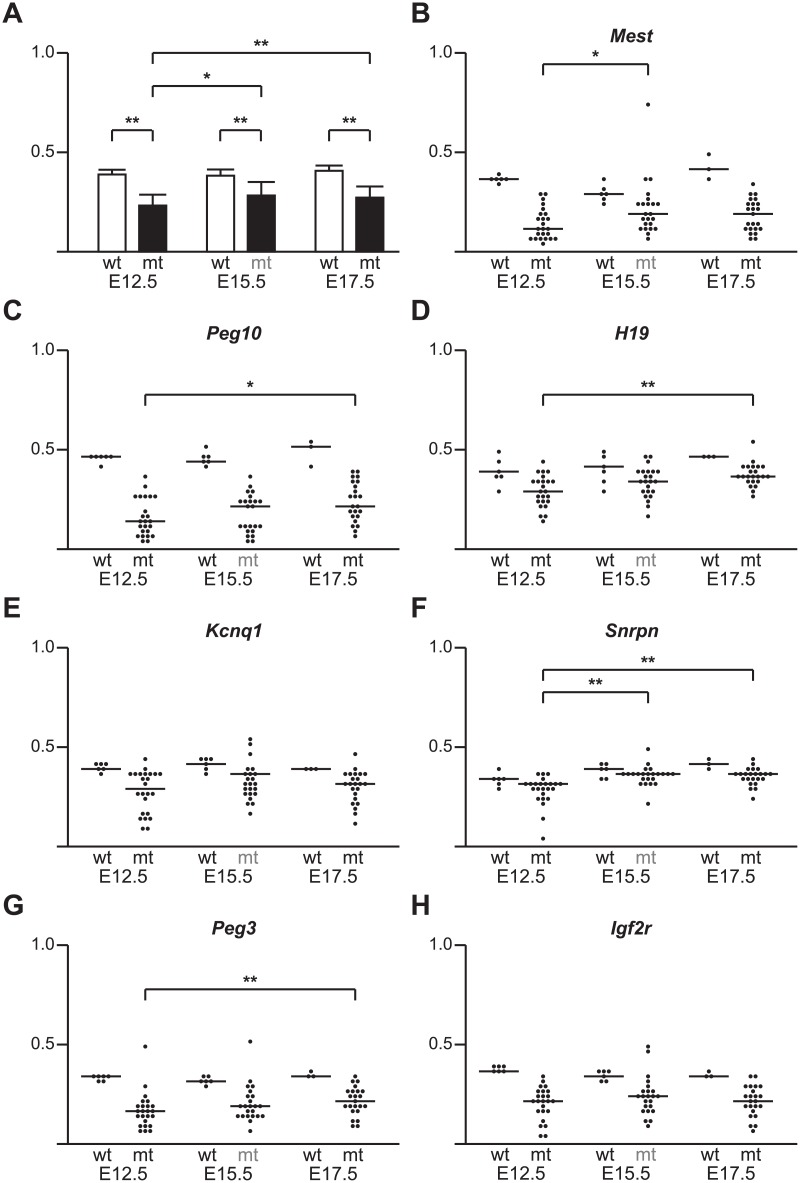
Imprinted gDMD methylation levels in wild-type (wt) and DNMT1o-deficient (mt) placentas across mid gestation (E12.5, E15.5 and E17.5). (A) Bar graphs showing average mean and standard deviation of total imprinted gDMD methylation of wt (open bars) and mt (filled bars) across mid-gestation. (B-F) Binned scatter plot showing individual wt and mt placentas across mid-gestation and the sample mean for the following imprinted gDMDs: (B) *Mest*, (C) *Peg10*, (D) *H19*, (E) *Kcnq1*, (F) *Snrpn*, (G) *Peg3* and (H) *Igf2r*. Small brackets indicate significant differences between gestational age matched sample populations of wt and mt gDMD methylation medians. Larger brackets indicate significant differences between mutant gDMD methylation medians at different gestational ages. * (P<0.01), and **(P<0.001) denote significant differences of mutant median imprinted gDMD methylation compared to wild type, or between gestational ages of mutant sample population by the Rank-sum test.

To understand the role of imprinted methylation on the wide-range of placental abnormalities seen in the *Dnmt1* ^*Δ1o*^ model, we first measured DNA methylation at 15 imprinted gDMDs at three times during the latter half of gestation (S1 Table). We calculated the average methylation fraction across 12 non-redundant gDMD EpiTYPER amplicons for both wild-type and mutant samples at each time point. Methylation was reduced in DNMT1o-deficient placentas at E12.5, E15.5 and E17.5 (Fig 1A). At E12.5 there was a significant decrease in the average methylation across all gDMDs (P<0.001) from 0.388 for wild-type to 0.232 for mutant placentas. In a collection of 23 E15.5 DNMT1o-deficient placentas, the average gDMD methylation was 0.283, significantly lower than the wild-type average of 0.382 (P<0.001). Similarly in a collection of 24 E17.5 placentas average gDMD methylation was 0.272, significantly lower than the wild-type average of 0.407 (P<0.001). There was a trend toward mutants approaching wild-type levels of imprinted gDMD methylation levels as gestation progressed; average gDMD methylation increased from E12.5 to E15.5 (P<0.01) and from E12.5 to E17.5 (P<0.001) but not from E15.5 to E17.5 (not significant). These findings show that total gDMD methylation levels in DNMT1o-deficient placentas do not remain constant across gestation but rather resolve to more normal levels, suggesting selection against low gDMD methylation epigenotypes that do not support placental development and function.

As expected, most individual imprinted gDMDs in DNMT1o-deficient placentas were significantly less methylated than gDMDs in wild-type placentas (Fig 1B–1H and S1 Fig). Concomitant with the increasing average imprinted gDMD methylation from E12.5 to E15.5 in DNMT1o-deficient placentas (Fig 1A), we observed a steady reduction in the range of methylation among the examined placentas for many but not all individual gDMDs (Fig 1B–1H and S1 Fig). Based on this, we were able to define three distinct temporal patterns of methylation. A group of five gDMDs had higher average methylation at E15.5 than at E12.5 (*Mest*, *Snrpn*, *Dlk1*.A, *Dlk1*.B, and *Nespas*.B; P<0.025; Fig 1B and 1F, S1C, S1D and S1F Fig). Other gDMDs had a more gradual increase in methylation from E12.5 to E17.5 (*Peg10*, *H19* and *Peg3*; P<0.025; Fig 1C, 1D and 1G). Five gDMDs comprise a third gDMD class that did not significantly change their average methylation across gestation in DNMT1o-deficient placentas (*Kcnq1*, *Igf2r*, *Plagl1*, *Nespas*.A, *Nespas*.B, *Impact*.A and *Impact*.B; Fig 1E and 1H, S1B and S1E–S1H Fig ). Out of the three imprinted gDMDS for which duplicate adjacent EpiTYPER amplicons were selected (*Dlk1*, *Impact* and *Nespas*), only *Nespas* showed a discordant trend with *Nespas*.A not differing between gestational cohorts and *Nespas*.B transitioning to higher average methylation between E12.5 and E15.5 (S1C–S1H Fig). Additionally the *Grb10* gDMD displayed opposing changes from E12.5 to E15.5 and E15.5 to E17.5, and did not significantly differ between E12.5 and E17.5 (S1A Fig). Three putative imprinted gDMDs were examined in E12.5 and E15.5 cohorts (*Commd1*, *Nnat* and *Nap1l5*), and only *Commd1* had a significant difference between wild-type and the *Dnmt1* ^*Δ1o*^ mutant average methylation levels (S2 Fig). In DNMT1o-deficient placentas *Commd1* remained variable and did not differ between E12.5 and E15.5. Overall, the observed trends in gDMD methylation during gestation suggest that there are strong biological influences blocking the loss of imprints at specific gDMDs during mid-gestation.

**Fig 2:**
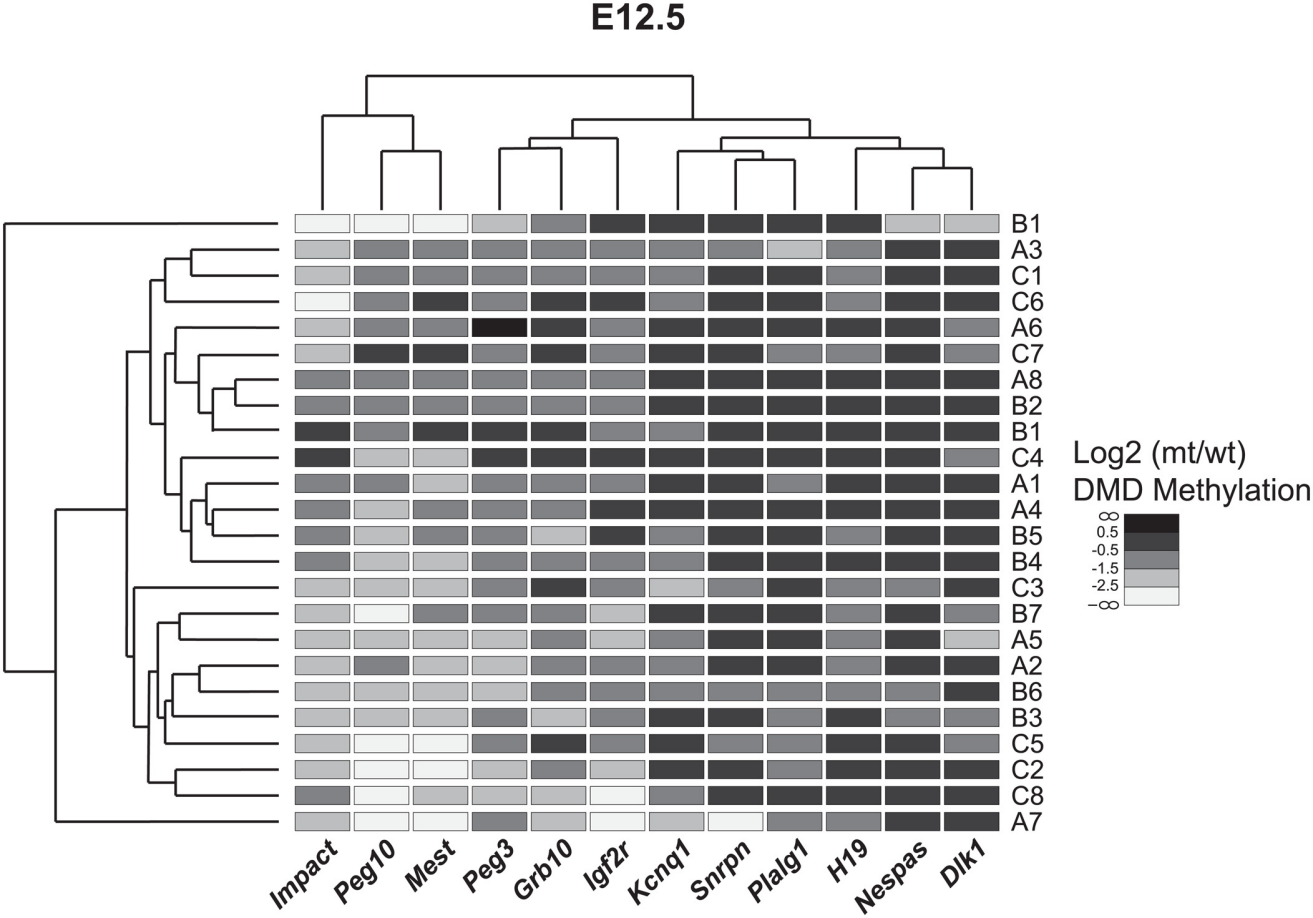
Hierarchical clustering of 24 E12.5 DNMT1o-deficient placentas based on gDMD methylation. Data is shown as the log2 transformed ratio of mt:wt gDMD methylation. The heat map displays normally methylated gDMDs as dark boxes whereas loss of methylation is indicated by lighter shades. The upper and side dendrograms display linkage between imprinted gDMDs and DNMT1o-deficient samples respectively. Imprinted gDMDs are labeled across the bottom axis. DNMT1o-deficient samples are labeled down the right hand side by cohort litter (Letters A-C) and conceptus (Numbers 1–8).

The spectrum of methylation among 12 gDMDs for each individual E12.5 DNMT1o-deficient placenta is displayed in the form of a heat map clustergram (Fig 2). Among the 24 E12.5 placentas represented in this manner, the majority of placentas have a unique gDMD methylation profile not found in other placentas, although there are a few cases of high similarity. For example placentas A8 and B2 have identical gDMD methylation profiles. Placentas A4 and B5 differ only at *Grb10*, *Kcnq1* and *H19* gDMDs, and placentas A3 and C1 are unique at only the *Plagl1* gDMD. Clustering of the gDMDs at E12.5 indicate the genetically linked *Peg10* and *Mest* gDMDs as well as the linked *Kcnq1* and *Snrpn* gDMDs vary in conjunction. Although there is a trend toward more normal gDMD methylation levels at E15.5 and E17.5, each DNMT1o-deficient placenta at these stages still has a unique imprinted epigenotype (S3 and S4 Figs). These comparisons among placentas across the latter half of gestation point out the intrinsic power of the *Dnmt1* ^*Δ1o*^ maternal effect model to produce diverse and abnormal patterns of imprinted gDMD methylation.

### Fetal viability is associated with gDMD methylation

DNMT1o-deficient placentas were collected at three gestational time points (E12.5, E15.5 and E17.5) and their phenotypic and epigenetic abnormalities described. A significant number of intact and viable placentas were associated with a deceased fetus at each time point. At E12.5 litter sizes of *Dnmt1* ^*Δ1o/ Δ1o*^ dams were consistent with wild-type litters (average of eight conceptuses), although slightly more than half of the conceptuses contained dead embryos (Table 1). On average smaller numbers of conceptuses were found in litters from *Dnmt1* ^*Δ1o/ Δ1o*^ females at E15.5 and E17.5. At these two later gestational times however, we recovered a greater percentage of conceptuses with an intact placenta and live fetus (Table 1). All placentas that were not obviously necrotic produced intact genomic DNA for methylation analysis whether or not they were associated with a live fetus.

**Table 1:** Viability of mid-gestation DNMT1o-deficient conceptuses. Viability of litters collected at mid-gestation in this study.

Gestational Age (dpc)	# Litters	#Placentas(a)	#Live Embryos(b)
E12.5	3	24	10
E15.5	4	23	11
E17.5	5	23	14

a) Only intact (non-necrotic) placentas were counted. b) Embryo viability based on presence of active circulation. DPC—days post copulation.

Logistic regression was used to identify those imprinted gDMDs that exerted the greatest influence on fetal viability at E12.5 through placental imprinting (Table 2). We report the logistic regression coefficient (logit) as a measure of the effect of DMD methylation levels on the odds ratio of fetal survival. A positive association was discovered between *Peg10* gDMD methylation and fetal viability at E12.5 (P<0.05) indicating that placentas with loss of the *Peg10* methylation imprint are less likely to support a viable fetus. A negative association between *Nnat* gDMD methylation and fetal viability was observed (P<0.05). The only significant association identified between imprinted gDMD methylation and fetal viability at either E15.5 or E17.5 was a negative association between *Nespas*.B gDMD methylation and viability at E15.5 (P<0.05; S3 and S4 Tables). These findings suggest that in the context of the *Dnmt1* ^*Δ1o*^ maternal effect mouse model, nearly normal *Nnat* and *Nespas* imprinting may decrease viability.

**Table 2:** Bivariate regression analysis of E12.5 DNMT1o-deficient placentas based on gDMD methylation and placental phenotypes. Only significant (P<0.05) associations established by bivariate regression analysis between dependent placental phenotypes and independent imprinted gDMD methylation values are shown. Regression coefficient is either the logit (log odds ratio) for logistic regression for fetal viability, or the linear regression coefficient (β) for all other variables.

Placental Phenotype	Imprinted gDMD	Regression Coefficient	Significance (P-Value)
Fetal Viability	*Peg10*	2.17	3.25E-02
	*Nnat*	-2.49	4.12E-02
Placental Weight (mg)	*Nespas*.A	-127.5	6.21E-04
Spongiotrophoblast Central Volume (mm3)	*Nespas*.A	-3.99	1.41E-05
	*Nespas*.B	-2.65	3.54E-02
	*H19*	-1.7	4.08E-02
Labyrinth Central Volume (mm3)	*Peg10*	3.58	3.12E-02
	*Nnat*	-7.72	2.82E-04
Trophoblast Giant Cell Count (#/section)	*Kcnq1*	-508	1.87E-05
	*Snrpn*	-674	2.31E-04
	*Plagl1*	-438	1.97E-02
	*Nespas*.B	-575	4.38E-02

### Placental abnormalities are associated with loss of gDMD methylation

**Fig 3:**
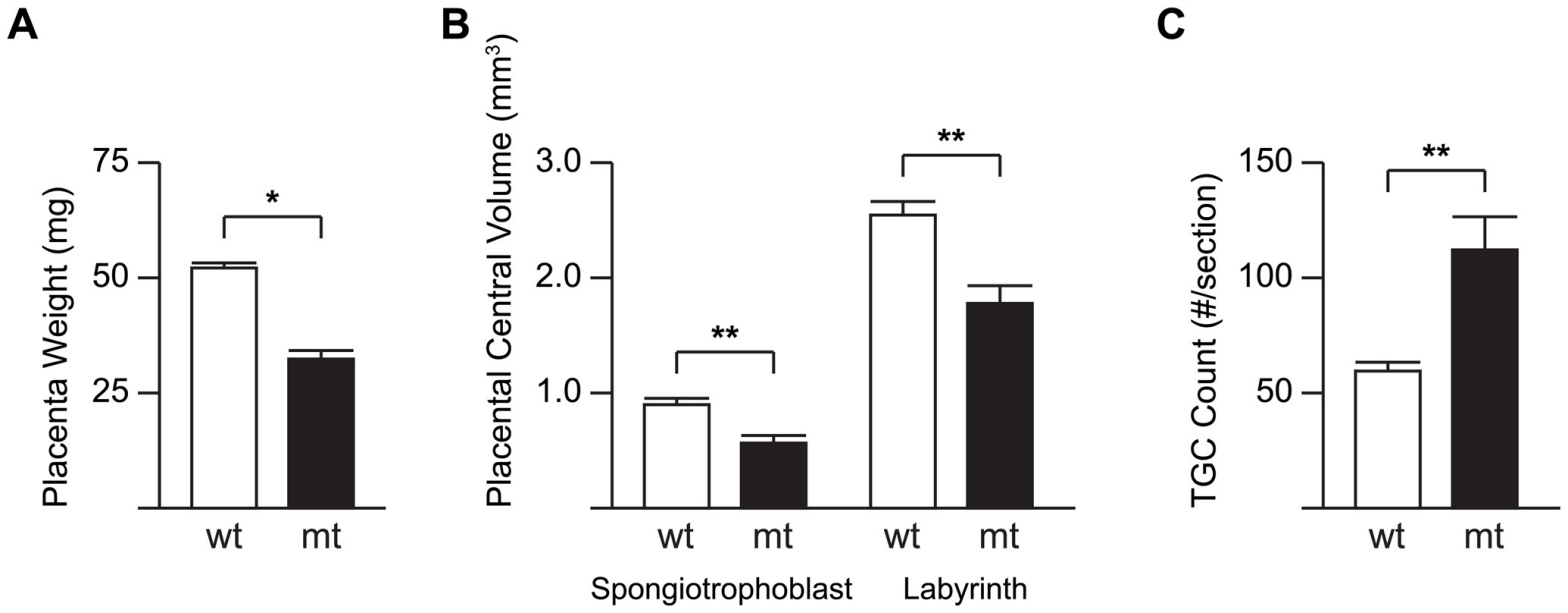
Phenotypic comparison of wild-type (wt) and DNMT1o-deficient (mt) placentas at E12.5. (A) Measurements of wet placenta weight, (B) Spongiotrophoblast and Labyrinth central volume, and (C) the number of TGCs per slide of a cohort of wt and mt placentas are displayed as open and filled bars respectively. Data are plotted as mean +SEM. *(P<0.05) and **(P<0.005) denote significant differences between wt and mt averages by the Rank-sum test.

DNMT1o-deficient and wild-type placentas differed in many ways at E12.5, particularly in weight, central spongiotrophoblast volume, central labyrinth volume and number of TGCs (Fig 3). There was a trend toward decreased placental weight in DNMT1o-deficient placentas (P<0.05; Fig 3A). Additionally there were significant decreases in measured central labyrinthine volume (P<0.005; Fig 3B) and central spongiotrophoblast volume (P<0.005; Fig 3B) in the DNMT1o-deficient placentas compared to wild-type controls. A marked increase in the number of TGCs per central section was measured in the E12.5 DNMT1o-deficient placentas compared to wild-type controls (P<0.01; Fig 3C). These findings are in line with our previous reports of distorted placental layer development at E9.5 (30).

To determine the effects of fetal viability and sex on placental phenotypes in the *Dnmt1* ^*Δ1o*^ maternal effect mouse model we compared live/dead and male/female mutant and wild-type cohorts (S5 and S6 Figs). We compared the phenotypes of DNMT1o-deficient placentas that harbored live and dead fetuses and found that those placentas that did not support a viable fetus had less labyrinth volume than those that did support a live fetus. In a sex comparison of DNMT1o-deficient placentas we discovered that female placentas on average had smaller central labyrinth volumes than mutant males (P<0.05; S6B Fig). In addition, DNMT1o-deficient females had significant differences from wild-type counterparts at all measured phenotypes whereas mutant males only differed from wild-type males in labyrinth central volume and TGC number. These results are in line with previously reported exacerbated early placental phenotypes in female offspring from the *Dnmt1* ^*Δ1o*^ maternal effect mouse model [32].

The variability in phenotypic metrics observed at E15.5 was smaller than that seen at E12.5. Specifically, at E15.5 neither spongiotrophoblast central volume nor labyrinth central volume phenotypic metrics significantly differed between wild-type and mutant cohorts (S7C Fig). However, there was an increase in both placental and fetal weights in DNMT1o-deficient placentas compared to gestational age matched controls (S7A and S7B Fig). We found in our comparison of live and dead mutants (S8 Fig) that viable DNMT1o-deficient placentas and fetuses were overgrown (P<0.005; S8A and S8B Fig). Mutant female placentas weighed more than wild-type females (P<0.05) but males did not differ from their wild-type counterparts (S9A Fig). Furthermore we analyzed placental and fetal weights at E17.5 and found that those placentas recovered at E17.5 were overgrown (P<0.005; S10A Fig). Conceptuses supporting live fetuses harbored heavier placentas (P<0.005) and fetuses (P<0.005) than those that were not viable (S11A and S11B Fig). Both male and female mutant placentas were heavier than wild-type controls (P<0.005 and P<0.05; S12A Fig).

Because of the broader range of abnormal gDMD methylation, histomorphological abnormalities and effects on fetal viability at E12.5 we focused our detailed phenotype-epigenotype regression analysis on the E12.5 time point. Bivariate linear regression analysis was used to determine which imprinted gDMDs underlie the observed E12.5 placental abnormalities. The most significant (P<0.05) gDMD associations for each phenotype are displayed in Table 2 and S13 and S14 Figs We report the regression coefficient (β) as the change in phenotype associated with modulation of the gDMD methylation fraction (0 to 1.0). Placenta weight is negatively associated with gDMD methylation at *Nespas*.A (Table 2 and S13A Fig) although not at *Nespas*.B. For each 1% decrease in *Nespas*.A gDMD methylation (0.01 methylation fraction) placental weight increased by a corresponding 1.275 milligrams (95% CI: 0.648, 1.902). Spongiotrophoblast volume was negatively associated with both analyzed *Nespas* regions as well as the *H19* gDMD (Table 2 and S13B–S13D Fig). Each 1% decrease in gDMD methylation at *Nespas*.A, *Nespas*.B and *H19* increased spongiotrophoblast volume by 0.0399 (95% CI: 0.0258, 0.054), 0.0266 (95% CI: 0.035, 0.0497) and 0.0170 (95% CI: 0.0017, 0.0323) mm^3^ respectively.

**Fig 4:**
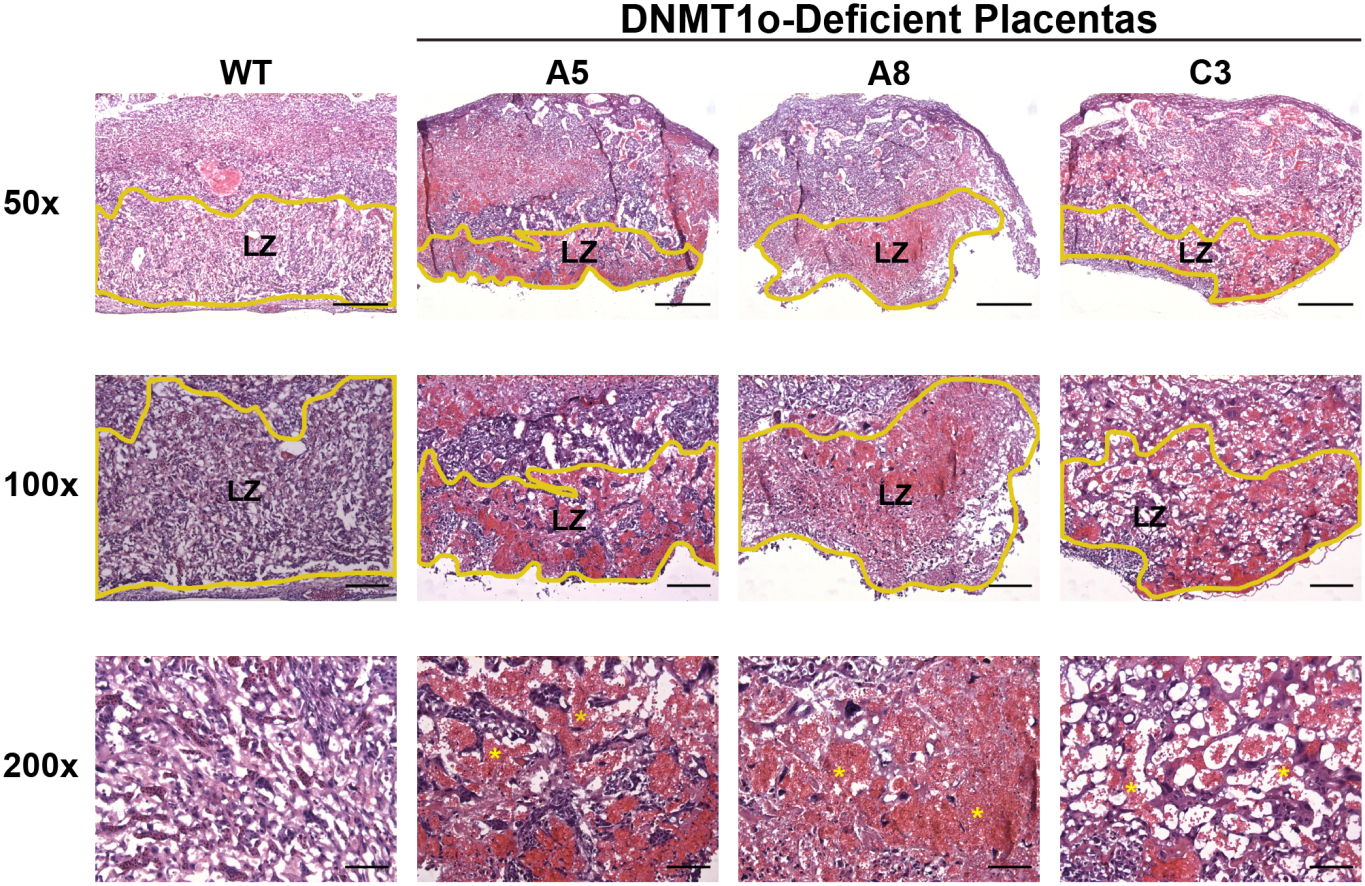
Histology of hematoxylin and eosin (H&E) stained labyrinth of one wild-type (wt) three DNMT1o-deficient low-*Peg10* gDMD methylation placentas. The scale bars for 50X, 100X and 200X magnification are 500, 200 and 100 μm respectively. Yellow lines in 50x and 100x magnification images outline the labyrinthine zone (LZ).

Linear regression analysis revealed a strong association between *Peg10* gDMD methylation and labyrinth volume (Table 2, S13E Fig). Diminishment of *Peg10* gDMD methylation by 1% corresponds to a 0.0217 (95% CI: 0.002, 0.066) mm^3^ decrease in labyrinthine central volume. Labyrinth structures in three DNMT1o-deficient placentas with low *Peg10* gDMD methylation are shown in Fig 4. Labyrinths in these samples are noticeably smaller, disorganized and hemorrhagic. Notably, methylation of the *Nnat* gDMD is negatively associated with labyrinth volume (Table 2 and S13F Fig), counter to the observed trend of decreased labyrinth in DNMT1o-deficient placentas; a 1% decrease in *Nnat* gDMD methylation resulting in a 0.0249 (95% CI: 0.043, 0.111) mm^3^ increase in labyrinth central volume.

**Fig 5:**
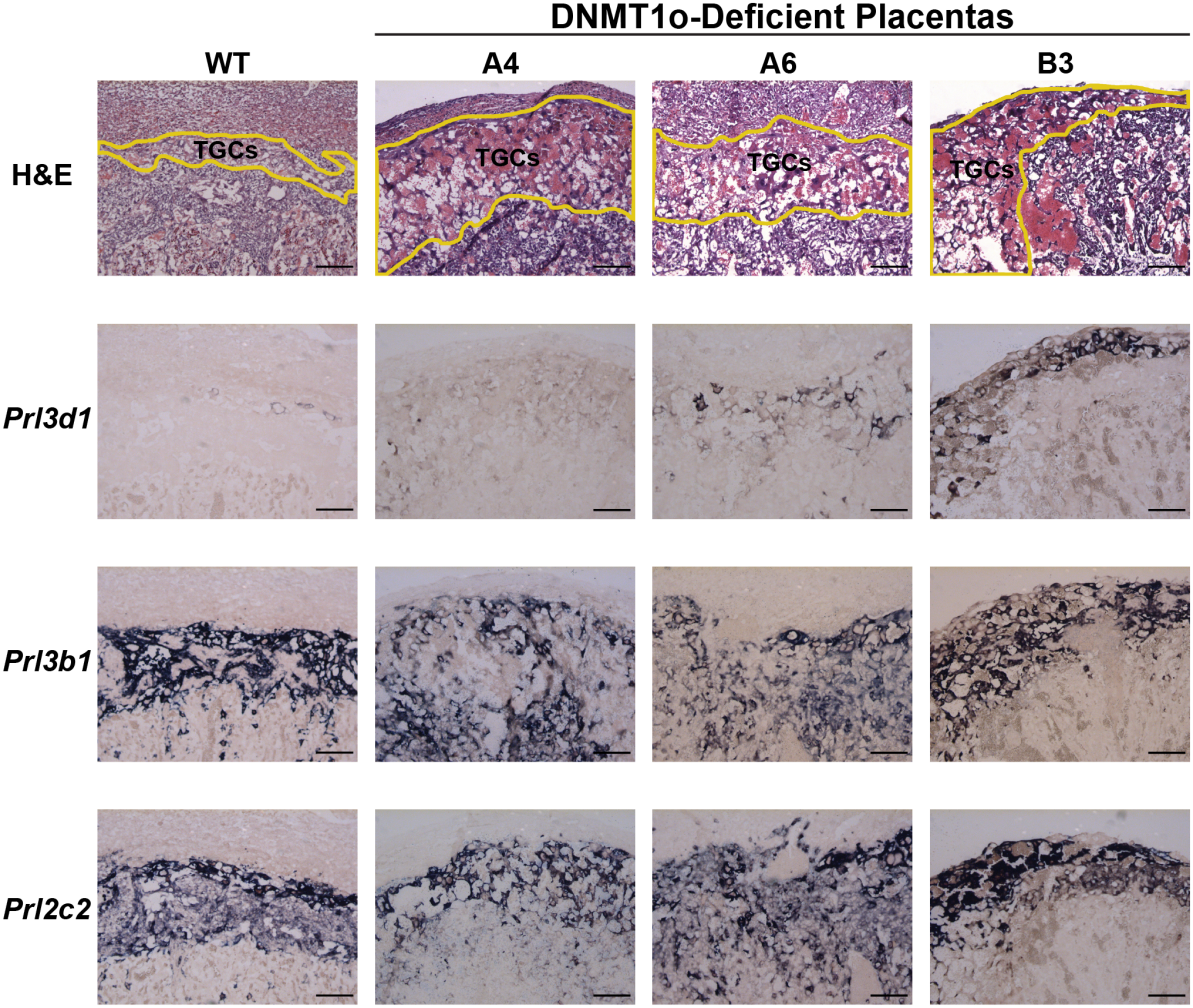
In situ hybridization analysis of TGCs in E12.5 wild-type and DNMT1o-deficient placentas with low *Kcnq1* gDMD methylation. All images were taken at 100X magnification. The scale bar is 100μm. Yellow lines delineate the layer containing trophoblast giant cells (TGCs) in the top row displays histology of hematoxylin and eosin (H&E) stained sections. *ISH* for the prolactin gene family members *Prl3d1*, *Prl3b1* and *Prl2c2* on adjacent sections to H&E are shown in the lower three rows.

Bivariate regression analysis revealed a significant negative association between *Kcnq1* gDMD methylation and accumulation of TGCs (Table 2 and S14A Fig). A 1% decrease in *Kcnq1* gDMD methylation corresponds to an increase of 5.08 (95% CI: 3.25, 6.91) TGCs per histological section. Representative H&E and *ISH* stained histological sections of wild-type and DNMT1o-deficient placentas with very low *Kcnq1* gDMD methylation and pronounced expansion of parietal TGCs bordering the spongiotrophoblast are displayed in Fig 5. Positive *ISH* staining for the pan-TGC transcripts proliferin (*Plf*; *Prl2c2*) and Prolactin-2 (*Pl2*; *Prl3b1*) was observed in both parietal TGCs and spongiotrophoblast layers (Fig 5). Intriguingly, the early TGC marker Prolactin-1 (*Pl1*; *Prl3d1*) was ectopically expressed in the parietal TGCs of DNMT1o deficient placentas with low *Kcnq1* gDMD methylation, whereas it should be restricted to TGCs embedded within maternal spiral arteries by E12.5.

DNA methylation at the genetically linked *Snrpn* gDMD (both *Kcnq1* and *Snrpn* gDMDs are on mouse chromosome 7) also inversely associated with TGC accumulation (Table 2 and S14B Fig). For every 1% decrease in *Snrpn* gDMD methylation there is a corresponding increase of 6.74 (95% CI: 3.73, 9.75) TGCs per section. A weaker inverse association between both *Plagl1* and *Nespas*.B gDMD methylation and TGC number was also identified (Table 2 and S14C and S14D Fig). Decreases of 1% methylation at *Plagl1* and *Nespas*.B modulate an increase in TGCs per section of 4.38(95% CI: 0.970, 7.79) and 5.75(95% CI: 0.500, 11.0) respectively. Imprinted DNA methylation at the *Peg3* gDMD, which like *Kcnq1* and *Snrpn* is a maternally derived methylation imprint on mouse chromosome 7, was not significantly associated with TGC accumulation (P = 0.40). Linear regression model building confirmed the major gDMD methylation influence of TGC accumulation to that of just the *Kcnq1* and *Snrpn* gDMDs (S2 Table).

To determine if there were any phenotype-epigenotype assocations at E15.5 and E17.5 we performed bivariate linear regression. At E15.5 spongiotrophoblast central volume inversely associated with *Impact*.B and *Mest* methylation (P<0.05; S3 Table). Each 1% decrease in *Impact*.B and *Mest* methylation increased spongiotrophoblast central volume by 0.0717 (95% CI: 0.043, 0.1004) and 0.0449 (95% CI: 0.0275, 0.0623) mm^3^ respectively. Using a relaxed significance threshold we found only three meaningful phenotype-epigenotype associations at E17.5 (P<0.075; S4 Table). Placental weight was positively associated with *Dlk1*.A methylation: each 1% decrease in *Dlk1*.A methylation corresponded to a 1.166 (95% CI: 0.591, 1.741) milligram decrease in placental weight. Fetal weight was associated with placental methylation at the *Igf2r* and *Mest* gDMDs: for each 1% decrease in *Igf2r* and *Mest* methylation fetal weight decreased by 20.40 (95% CI: 11.17, 29.63) and 16.81 (95% CI: 8.05, 25.57) milligrams respectively.

## Discussion

Results presented here and in our prior work [31] provide unequivocal evidence in support of the importance of imprinted gDMD methylation during placental development. In this study we ascribed placental function for gDMDs in two ways: by identifying nearly normal gDMD methylation in DNMT1o-deficient placentas; and by correlating highly variable gDMD methylation with placental phenotypes at E12.5. We expected total wild-type placental gDMD methylation to be approximately 50%, but found the wild-type average to be just under 40% at each time point. These results are consistent with the slightly lower levels of gDMD methylation found in control placentas than embryos in prior studies [32]. Our results show a large range of methylation across individual gDMDs with *Peg3* (32.7%) on the low end and *Dlk1*.A (57.7%) on the high end in wild-type E12.5 samples. Based on our understanding of DNMT1o action it is predicted that on average a 50% loss of methylation at each gDMD should be observed in cohorts of DNMT1o-deficient placentas [3–5]. However, we found that a few imprinted gDMDs were nearly normally methylated at E12.5 (i.e. *H19*, *Dlk1*.A, *Dlk1*.B and *Nespas*.B; Fig 1D, S1C, S1D and S1F Fig) and E15.5 (ie. *H19*, *Snrpn*, *Dlk1*.A, *Dlk1*.B, and *Nespas*.B; Fig 1D and 1F, S1C, S1D and S1F Fig). These findings suggest that many epigenotypes with these gDMDs poorly methylated may be incompatible with early trophoblast survival and/or proliferation resulting in selection against specific epigenotypes at the cellular and organismal level.

Interrogation of the association of gDMD methylation and placental phenotypes by regression analysis confirmed the importance of gDMD methylation in placental development and function. Herein, significant associations were observed between diminished imprinted methylation of the variable gDMDs (*Peg10*, *Kcnq1*, *H19* and *Nespas*) and specific placental phenotypes in DNMT1o-deficient E12.5 placentas (Table 2; S13 and S14 Figs). Importantly, this approach using the *Dnmt1* ^*Δ1o*^ maternal effect model to gain insight into the role of imprinted genes in placental development and function is fundamentally different in two significant ways from genetic approaches that inactivate either single imprinted genes or remove imprinting control centers. First, the *Dnmt1* ^*Δ1o*^ maternal effect model produces epigenetic mutant offspring with loss of gDMD methylation, while retaining the genetic sequence of ICRs and imprinted genes. Second, the *Dnmt1* ^*Δ1o*^ maternal effect model produces broadly variable methylation effects across many gDMDs. This permits gDMD methylation to be treated as a continuous variable in a quantitative trait analysis, thus revealing strong associations between loss of methylation at particular gDMDs and histo-morphological placental phenotypes. The recognition of these associations offers new insights into the integral role of genomic imprints in placenta development.

### *Peg10* Viability and Labyrinth Phenotypes

A strong association was observed between loss of *Peg10* gDMD methylation and decreased fetal viability and labyrinth volume at E12.5 (Table 2 and S13 Fig). Most placentas with loss of *Peg10* gDMD methylation and decreased labyrinth volume were unable to support fetal development. We interpret these associations, and the gradual trend toward normal *Peg10* gDMD methylation levels from E12.5 to E17.5 (Fig 1C), as a progressive requirement for *Peg10* methylation to sustain fetal viability during later gestation. The decreasing *Peg10* gDMD methylation variability and lack of phenotypic association at E15.5 and E17.5 could be explained by selection against certain low *Peg10* gDMD methylation epigenotypes. The gDMD methylation epigenotype of placentas with low *Peg10* methylation at E12.5 is different than the epigenotype of placentas with low *Peg10* gDMD methylation recovered at E15.5 and E17.5 (Fig 2, S4 and S7 Figs). The combination of low *Peg10* gDMD methylation (<50% wild-type level) plus low *Dlk1*, *Kcnq1*, *Nespas* or *Snrpn* gDMD methylation (<50% wild-type) was observed at E12.5 (samples A5, A7, B1 and C3; Fig 2) but does not occur in any DNMT1o-deficient placentas at either E15.5 or E17.5 (S3 and S4 Figs). In summary, our analysis of DNMT1o-deficient placentas reveals a novel link between placentas with low *Peg10* gDMD methylation, poor labyrinth development and the inability to sustain fetal development.

We found a strong linkage between *Mest* and *Peg10* gDMD methylation at E12.5 and E17.5 (Fig 2 and S7 Fig). This was expected given the proximity of the two gDMDs on mouse chromosome 6, however *Mest* did not show significant associations with early placental phenotypes in this study. This observation does not preclude a role for *Mest* later in gestation, and in fact we previously discovered a link between loss of *Mest* gDMD methylation and placental lipid accumulation at E17.5 [35]. In our current study we found an inverse association between *Mest* gDMD methylation and spongiotrophoblast volume at E12.5 (S3 Table) and a positive association between *Mest* and fetal weight at E17.5 (S4 Table). We suggest that *Mest* and *Peg10* gDMDs may exert their influence on placental development in a serial manner; loss of *Peg10* gDMD methylation impairs labyrinth development early in gestation, which predisposes these placentas to metabolic abnormalities associated with lost *Mest* gDMD methylation later in gestation.

The lethality and labyrinth failure in DNMT1o-deficient placentas with low *Peg10* gDMD methylation is similar to the phenotype observed in *Peg10* null mice [27]. Although the expected result of loss of *Peg10* gDMD methylation is increased Peg10 expression, we previously failed to detect significant changes in *Peg10* expression in DNMT1o-deficient placentas at any time point between E9.5 and E17.5 [31]. However, we did previously observe a significant increase in *Sgce* and *Pon2* expression in late gestation DNMT1o-deficient placentas [31]. It is difficult to correlate gDMD methylation with imprinted gene expression in *Dnmt1* ^*Δ1o*^ maternal effect placentas because of the confounding factors of a mosaic model, cell-type expression biases and differential effects of loss of gDMD methylation. Based on our direct observation that partial loss of a maternally methylated *Peg10* imprint is detrimental to placental development, we suggest that strict monoallelic dosage of *Peg10*, and/or other imprinted genes within the *Peg10* imprinted cluster is critical for placental development.

### Loss of *Kcnq1* gDMD methylation and TGC expansion

Mouse chromosome 7 contains one paternally (*H19*) and three maternally (*Kcnq1*, *Snrpn* and *Peg3*) methylated gDMDs. Not surprisingly, we found that the methylation status of the *Kcnq1* and *Snrpn* gDMDs was linked at E12.5 in DNMT1o-deficient placentas (Fig 2). A strong association between DNA methylation at these two gDMDs and accumulation of TGCs was unearthed (Table 2 and S14 Fig). Based on our forward step-wise regression model (S2 Table) the combination of gDMD methylation levels of *Kcnq1* and *Snrpn* is the best predictor of TGC abundance. We speculate that the association between *Snrpn* methylation and TGC accumulation is a passive effect due to close linkage with the *Kcnq1* cluster and consistent with lack of known placental function for *Snrpn* [12]. The *in situ* staining of TGCs for *Prl3d1* in DNMT1o-deficient placentas, an early TGC marker, indicates that not only is proliferation altered but also TGC differentiation (Fig 5). The morphology of DNMT1o-deficient placentas with low *Kcnq1* gDMD methylation is similar to those described in null and hypomorphic *Ascl2* (a maternally expressed transcription factor and member of the *Kcnq1* cluster) mouse models in which expansion of TGCs was observed [14, 36]. It has previously been demonstrated that DNMT1o-deficient placentas have decreased expression of *Ascl2* [31, 37]

The accumulation of TGCs observed in the *Dnmt1* ^*Δ1o*^ maternal effect model shown herein is remarkably similar to placentas derived from *Dnmt3L* null mothers, which lack all maternal imprinted gDMD methylation [38, 39]. One mechanistic explanation of the TGC expansion that is common between the *Dnmt1* ^*Δ1o*^, *Dnmt3L* and *Ascl2* models is that a decrease in *Ascl2* expression (by gene deletion or loss of *Kcnq1* gDMD methylation) results in derepression of *Hand1*, a transcription factor that promotes differentiation of the ectoplacental cone and spongiotrophoblast into TGCs [40–42]. Loss of *Kcnq1* gDMD methylation in DNMT1o-deficient placentas has a distinct phenotype from paternal deletion of the *Kcnq1* ICR, which mimics a maternal (methylated) state with resulting increased maternal expression of *Ascl2*, *Phlda2*, and *Cdkn1c*, and growth restriction [43]. Regression analysis did not reveal meaningful associations between loss of *Kcnq1* gDMD methylation and placental overgrowth at E15.5 or E17.5 that might be expected based on targeted deletion mouse models of *Phlda2* and *Cdkn1c*, which exhibit pronounced placental overgrowth [15, 16]. Our findings taken together with prior research suggest that the imprinted gene *Ascl2* is a focal point for early placental development.

### Loss of *Nespas* and *H19* gDMD and spongiotrophoblast development

In addition to the effects of reduced *Peg10* and *Kcnq1* gDMD methylation discussed above, this study revealed weaker, but nonetheless significant, associations between loss of imprinted *Nespas* and *H19* gDMD methylation and increased spongiotrophoblast volume (Table 2 and S13B–S13D Fig). Although both *Nespas* gDMD amplicons assayed associated significantly with spongiotrophoblast expansion at E12.5 (Table 2, S13B and S13C Fig), an association was not observed at E15.5 (S3 Table), indicating this phenotype may resolve to a more normal one during development.

The observed association between loss of *H19* gDMD methylation and spongiotrophoblast expansion bordered the significant cutoff (P = 0.048, Table 2 and S13D Fig). *H19* gDMD methylation gradually increased from E12.5 to E17.5 in DNMT1o-deficient placentas, indicating selection against loss of imprinting at this cluster. Loss of methylation at the *H19* gDMD is expected to depress transcription of the growth factor *Igf2*. We previously described loss of *Igf2* expression in DNMT1o-deficient placentas at in E9.5 and E12.5 but found more normal levels at E15.5 and E17.5 [31]. It is known that *Igf2* is paternally expressed throughout the placenta, and that the placenta specific isoform (*Igf2P0*) is expressed exclusively in labyrinth syncytiotrophoblast [20, 21]. Paternal inheritance of either the *Igf2* null or *Igf2P0* null allele results in placenta with reduced spongiotrophoblast volume [21]. Based on this knowledge one explanation for the observed trend is that spongiotrophoblast is less dependent on IGF2 signaling than labyrinthine cell types, and may increase as an early compensatory mechanism to low placental *Igf2* expression. The association between *H19* gDMD methylation and spongiotrophoblast volume is not found at E15.5 reflecting the resolving of both *H19* methylation levels and spongiotrophoblast volume toward normal levels.

### Associations of placental phenotype and *Dlk1*, *Igf2r* and *Grb10* gDMD methylation not found

At the onset of this study we expected to find associations between imprinted DNA methylation at the *Dlk1* gDMD and labyrinth development, and between both the *Grb10* and *Igf2r* gDMDs and placental growth based on evidence from genetic models [23, 24]. In DNMT1o-deficient placentas imprinted DNA methylation at the *Dlk1* gDMD did not significantly differ from wild-type although it did increase across gestation (S1C and S1D Fig). This pattern is perhaps indicative of early selection against cellular epigenotypes with loss of *Dlk1* gDMD methylation during trophoblast differentiation and proliferation. We did not find associations between *Dlk1* gDMD methylation and placental phenotypes at E12.5 and E15.5, but did find a positive association between *Dlk1*.A methylation and placental weight at E17.5 (S4 Table), indicating loss of *Dlk1* methylation restricts placental growth. Although there was substantial variation in gDMD methylation at the *Igf2r* and *Grb10* gDMDs (Fig 1H and S1A Fig), we did not find direct associations between placental weight and gDMD methylation at either loci at E12.5 or E15. However, we discovered a positive relationship between *Igf2r* gDMD methylation and fetal weight at E17.5 (S4 Fig), a counter intuitive finding given that loss of *Igf2r* methylation should repress expression of this growth suppressor. Regression analysis failed to identify gDMDs responsible for the overgrowth of late gestation placentas and embryos but rather identified ones that promoted growth restriction. We interpret these results as evidence that in the context of the *Dnmt1* ^*Δ1o*^ mosaic loss-of-imprinting model, the mid to late gestation growth effects of the *Grb10* and *Igf2r* gDMDs may be obscured by epigenetic epistatic interactions with loss of imprinting at other prominent gDMDs within both placental and embryonic compartments. The clinically relevant dysregulation of placental and fetal growth associated with loss of imprinting previously highlighted (31) and confirmed herein is likely due to these complex interactions between imprinted regions. In contrast, the stronger associations between both *Peg10* and *Kcnq1* and E12.5 placental phenotypes were not occluded by confounding epistatic effects.

### *Commd1* is an imprinted gDMD in placenta but *Nnat* and *Nap1l5* are not

We measured the DNA methylation levels of three additional imprinted gDMDs (*Commd1*, *Nnat* and *Nap1l5*) in wild-type and DNMT1o-deficient E12.5 and E15.5 placentas (S2 Fig). The mouse genomic coordinates for these three gDMDs were previously established [2], but were not examined in placenta. EpiTYPER analysis showed that the *Commd1* gDMD was methylated at a level consistent with imprinting in wild-type placenta, which was then lost in DNMT1o-deficient placentas (S2A Fig). Both the *Nnat* and *Nap1l5* gDMDs showed a methylation pattern that was not indicative of imprinted gDMDs (S2B and S2C Fig). Both gDMDs also had higher methylation levels in wild-type placentas than other imprinted gDMDs tested (gDMD methylation fraction >0.7), and furthermore, neither gDMD lost methylation in DNMT1o-deficient placentas. We conclude that neither *Nnat* nor *Nap1l5* are imprinted gDMDs that are perpetuated from gametes to mature trophoblast lineages, and that although the *Commd1* gDMD is imprinted in the placenta, loss of imprinting at this loci is tolerated. Recent genome methylation studies have provided evidence that the *Nap1l5* but not the *Nnat* gDMD retains its imprinted status in the human placenta [44, 45].

## Conclusion

In summary, we have validated the epigenetic variability inherent in the *Dnmt1* ^*Δ1o*^ maternal effect model using a broad survey of imprinted gDMD methylation. We discovered a novel association between loss of imprinting at the *Peg10* loci and fetal viability and placental labyrinth maldevelopment. In addition we found a strong association between loss of imprinting at the *Kcnq1* cluster and TGC accumulation, validating prior genetic models. We conclude from the lack of *Dlk1* gDMD methylation variability at E12.5 that *Dlk1* has an essential early trophoblast function. This study highlights the direct epigenetic effects of loss of imprinting on placenta development. Our findings provide additional rationale to further dissect the *Peg10* and *Kcnq1* imprinting clusters for their roles in placental development.

## Supporting Information

S1 Fig Additional analysis of imprinted gDMD methylation levels in wild-type (wt) and DNMT1o-deficient (mt) placentas across mid gestation (E12.5, E15.5 and E17.5). Binned scatter plot showing individual wt and mt placenta across mid-gestation and the sample mean for the following imprinted gDMDs: (A) *Grb10*, (B) *Plagl1*, (C) *Dlk1*.A, (D) *Dlk1*.B, (E) *Nespas*.A, (F) *Nespas*.B, (G) *Impact*.A and (H) *Impact*.B. Brackets indicate significant differences between mutant gDMD methylation medians at different gestational ages. * (P<0.01) and **(P<0.001) denote significant differences of mutant median imprinted gDMD methylation compared to wild type, or between gestational ages of mutant sample population by the Rank-sum test.
(PDF)

S2 Fig Additional DNA methylation analysis of three prospective imprinted gDMDs in wild-type (wt) and DNMT1o-deficient (mt) placentas at two mid gestational ages (E12.5 and E15.5). Data displayed as binned scatter plots showing individual wt and mt placentas across mid-gestation and the sample mean for the following imprinted gDMDs: (A) *Commd1*, (B) *Nnat*, (C) and *Nap1l5*. No significant changes in gDMD methylation levels between mt cohorts at E12.5 and E15.5 were detected by the Rank-Sum test.
(PDF)

S3 Fig Hierarchical clustering of 23 E15.5 DNMT1o-deficient placentas based on gDMD methylation. Data is shown as the log2 transformed ratio of mt:wt gDMD methylation. The heat map displays normally methylated gDMDs as dark boxes whereas loss of methylation is indicated by lighter shades. The upper and side dendrograms display linkage between imprinted gDMDs and DNMT1o-deficient samples respectively. Imprinted gDMDs are labeled across the bottom axis. DNMT1o-deficient samples are labeled down the right hand side by cohort litter (Letters A-D) and conceptus (Numbers 1–8).
(PDF)

S4 Fig Hierarchical clustering of 23 E17.5 DNMT1o-deficient placentas based on gDMD methylation. Data is shown as the log2 transformed ratio of mt:wt gDMD methylation. The heat map displays normally methylated gDMDs as dark boxes whereas loss of methylation is indicated by lighter shades. The upper and side dendrograms display linkage between imprinted gDMDs and DNMT1o-deficient samples respectively. Imprinted gDMDs are labeled across the bottom axis. DNMT1o-deficient samples are labeled down the right hand side by cohort litter (Letters A-G) and conceptus (Numbers 1–8).
(PDF)

S5 Fig Phenotypic comparison of wild-type (wt) and DNMT1o-deficient (mt) live and dead placentas at E12.5. (A) Wet placenta weight, (B) Spongiotrophoblast and Labyrinth central volume, and (C) the number of TGCs per slide of wt, mt-live and mt-dead placental cohorts are displayed as white, black and gray bars respectively. Data are plotted as mean + SEM. *(P<0.05) and **(P<0.005) denote significant differences between wt, mt-live and mt-dead averages by the Rank-sum test.
(PDF)

S6 Fig Phenotypic comparison of wild-type (wt) male and female, and DNMT1o-deficient (mt) male and female placentas at E12.5. (A) Wet placenta weight, (B) Spongiotrophoblast and Labyrinth central volume, and (C) the number of TGCs per slide of wt-male, mt-male, wt-female and mt-female placental cohorts are displayed as white, black, light-gray and dark-gray bars respectively. Data are plotted as mean + SEM. *(P<0.05) and **(P<0.005) denotes significant differences between wt-male, wt-female, mt-male and mt-female averages by the Rank-sum test.
(PDF)

S7 Fig Phenotypic comparison of wild-type (wt) and DNMT1o-deficient (mt) placentas and fetuses at E15.5. (A) Wet placenta weight, (B) Wet fetal weight, and (C) Spongiotrophoblast and Labyrinth central volume of wt and mt cohorts are displayed as open and filled bars respectively. Data are displayed as mean + SEM. * (P<0.05) and **(P<0.005) denote significant differences between wt and mt averages by the Rank-sum test.
(PDF)

S8 Fig Phenotypic comparison of wild-type (wt) and DNMT1o-deficient (mt) live and dead placentas at E15.5. (A) Wet placenta weight, (B) Wet fetal weight, and (C) Spongiotrophoblast and Labyrinth central volume of wt, mt-live and mt-dead cohorts are displayed as white, black and gray bars respectively. Data are plotted as mean + SEM. *(P<0.05) and **(P<0.005) denote significant differences between wt, mt-live and mt-dead averages by the Rank-sum test.
(PDF)

S9 Fig Phenotypic comparison of wild-type (wt) male and female, and DNMT1o-deficient (mt) male and female placentas at E15.5. (A) Wet placenta weight, (B) Wet fetal weight, and (C) Spongiotrophoblast and Labyrinth central volume of wt-male, mt-male, wt-female and mt-female cohorts are displayed as white, black, light-gray and dark-gray bars respectively. Data are plotted as mean + SEM. *(P<0.05) and **(P<0.005) denotes significant differences between wt-male, wt-female, mt-male and mt-female averages by the Rank-sum test.
(PDF)

S10 Fig Comparison of wild-type (wt) and DNMT1o-deficient placental and fetal weights at E17.5. (A) Wet placental weights and (B) Wet fetal weights of wt and mt cohorts are shown as open and filled bars respectively. Data are displayed as mean +SEM. ** (P<0.001) by the Rank-Sum test.
(PDF)

S11 Fig Phenotypic comparison of wild-type (wt) and DNMT1o-deficient (mt) live and dead placentas at E17.5. (A) Wet placenta weight and (B) Fetal weight of wt, mt-live and mt-dead conceptuses are displayed as white, black and gray bars respectively. Data are plotted as mean + SEM. *(P<0.05) and **(P<0.005) denote significant differences between wt, mt-live and mt-dead averages by the Rank-sum test.
(PDF)

S12 Fig Phenotypic comparison of wild-type (wt) male and female, and DNMT1o-deficient (mt) male and female placentas at E17.5. (A) Wet placenta weight, and (B) Wet fetal weight, of wt-male, mt-male, wt-female and mt-female conceptuses are displayed as white, black, light-gray and dark-gray bars respectively. Data are plotted as mean + SEM. *(P<0.05) and **(P<0.005) denote significant differences between wt-male, wt-female, mt-male and mt-female averages by the Rank-sum test.
(PDF)

S13 Fig Linear regression plots of imprinted gDMD methylation versus placental phenotypic metrics in a cohort of E12.5 DNMT1o-deficient placenta. (A) Negative association between *Nespas*.A gDMD methylation and placental weight. (B) Negative association between *Nespas*.A gDMD methylation and spongiotrophoblast volume. (C) Negative association between *Nespas*.B gDMD methylation and spongiotrophoblast volume. (D) Negative association between *H19* gDMD methylation and spongiotrophoblast volume. (E) Positive association between *Peg10* gDMD methylation and labyrinth volume. (F) Negative association between *Nnat* gDMD methylation and labyrinth volume. R^2^ is unadjusted R-square value.
(PDF)

S14 Fig Linear regression plots of imprinted gDMD methylation TGC counts in a cohort of E12.5 DNMT1o-deficient placenta. (A) Negative association between *Kcnq1* gDMD methylation and TGC counts. (B) Negative association between *Snrpn* gDMD methylation and TGC counts. (C) Negative association between *Plagl1* gDMD methylation and TGC counts. (D) Negative association between *Nespas*.B gDMD methylation and TGC counts. R^2^ is unadjusted R-square value.
(PDF)

S1 Table Bisulfite PCR Amplicons and Primer Sequence and Coordinates. Forward and Reverse sequence tags attached to each primer pair for EpiTYPER methylation analysis. Genomic coordinates of imprinted gDMDs from the most recent mouse genome build (GRCm38). Imprinted gDMD amplicons in bold were those used to calculate average imprinted gDMD methylation fraction in Fig 1A.
(PDF)

S2 Table Stepwise forward linear regression analysis of associations between imprinted gDMD methylation and TGC accumulation in E12.5 DNMT1o-deficient placentas. N = 24, df = 21, model P-value = 1.54x10^-5^.
(PDF)

S3 Table Bivariate regression analysis of E15.5 DNMT1o-deficient placental methylation and phenotypes. Only significant (P<0.05) associations are shown. Regression coefficient is either the logit (log odds ratio) for logistic regression for fetal viability, or the linear regression coefficient (β) for all other variables.
(PDF)

S4 Table Linear regression analysis reveals association between imprinted gDMD methylation and placental and fetal weights at E17.5. Only associations with strong (P<0.075) are shown. The linear regression coefficient (β) is reported for both phenotypic variables.
(PDF)
