# Supplementary figures and images for: Partial Loss of Genomic Imprinting Reveals Important Roles for Kcnq1 and Peg10 Imprinted Domains in Placental Development
Source: PLoS One. 2015 Aug 4;10(8):e0135202. doi: 10.1371/journal.pone.0135202 (PMC4524636; doi:10.1371/journal.pone.0135202)

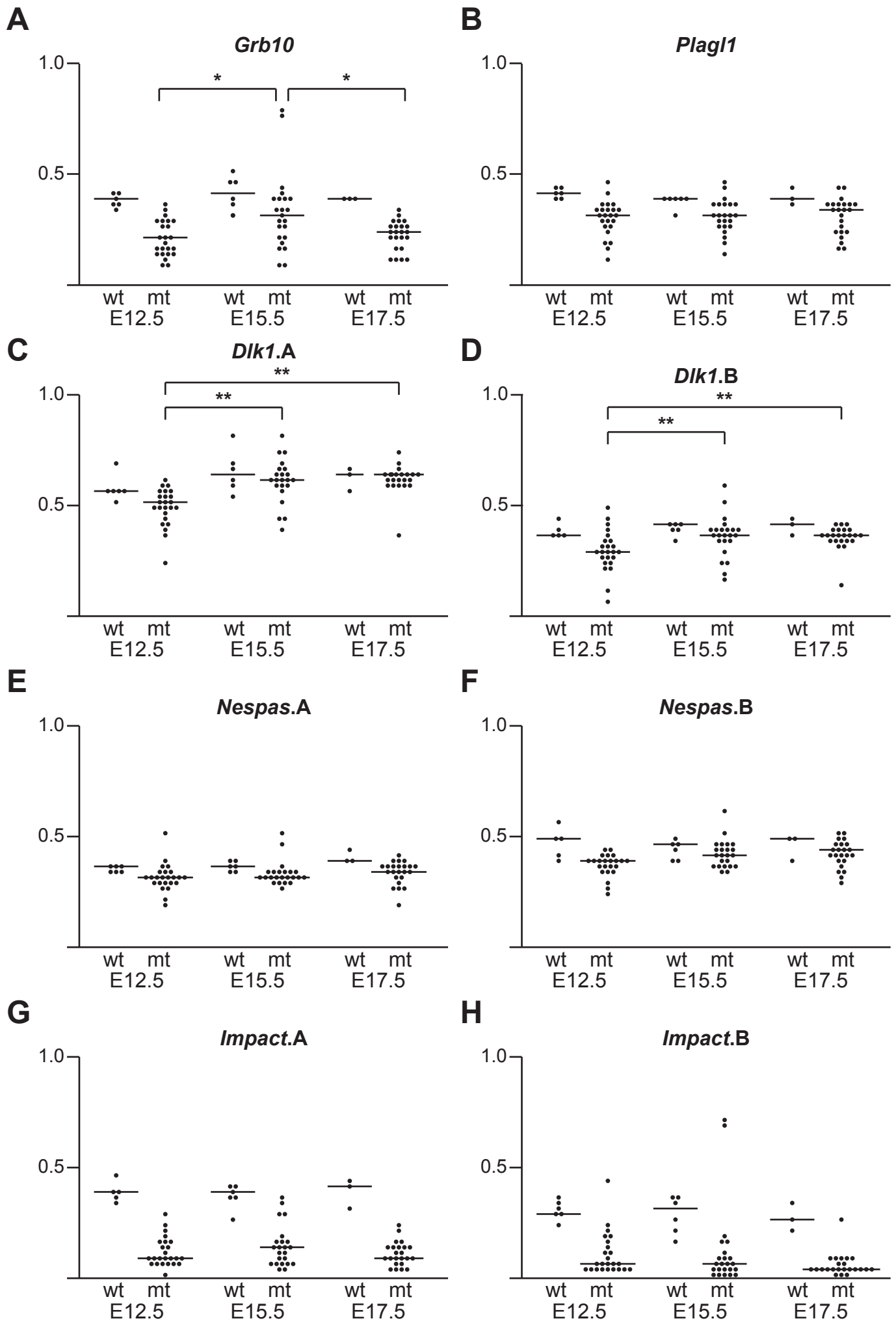

Supplement: S1 Fig — Binned scatter plot showing individual wt and mt placenta across mid-gestation and the sample mean for the following imprinted gDMDs: (A) Grb10, (B) Plagl1, (C) Dlk1.A, (D) Dlk1.B, (E) Nespas.A, (F) Nespas.B, (G) Impact.A and (H) Impact.B. Brackets indicate significant differences between mutant gDMD methylation medians at different gestational ages. * (P<0.01) and **(P<0.001) denote significant differences of mutant median imprinted gDMD methylation compared to wild type, or between gestational ages of mutant sample population by the Rank-sum test. (PDF) [file pone.0135202.s001.pdf]

**A**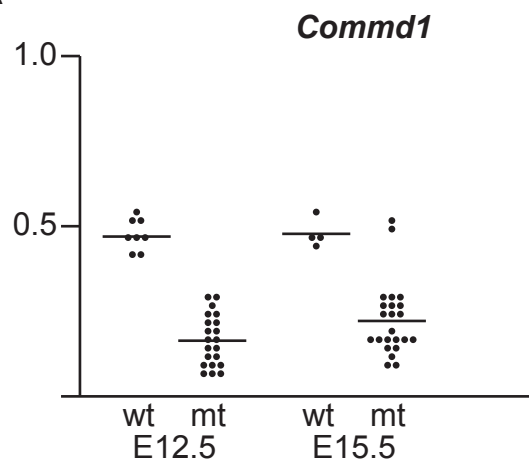**B**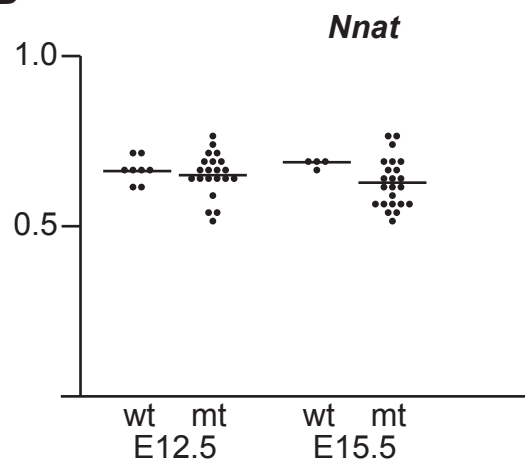**C**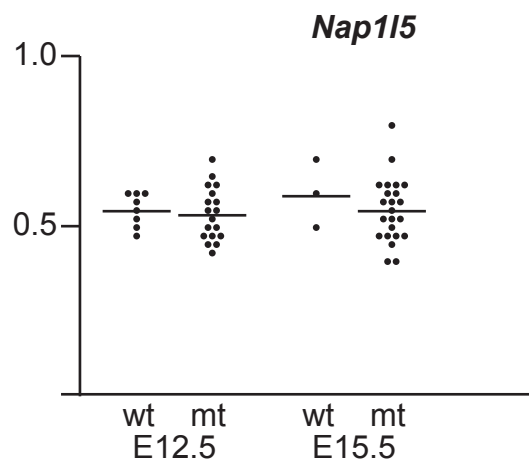

Supplement: S2 Fig — Data displayed as binned scatter plots showing individual wt and mt placentas across mid-gestation and the sample mean for the following imprinted gDMDs: (A) Commd1, (B) Nnat, (C) and Nap1l5. No significant changes in gDMD methylation levels between mt cohorts at E12.5 and E15.5 were detected by the Rank-Sum test. (PDF) [file pone.0135202.s002.pdf]

E15.5

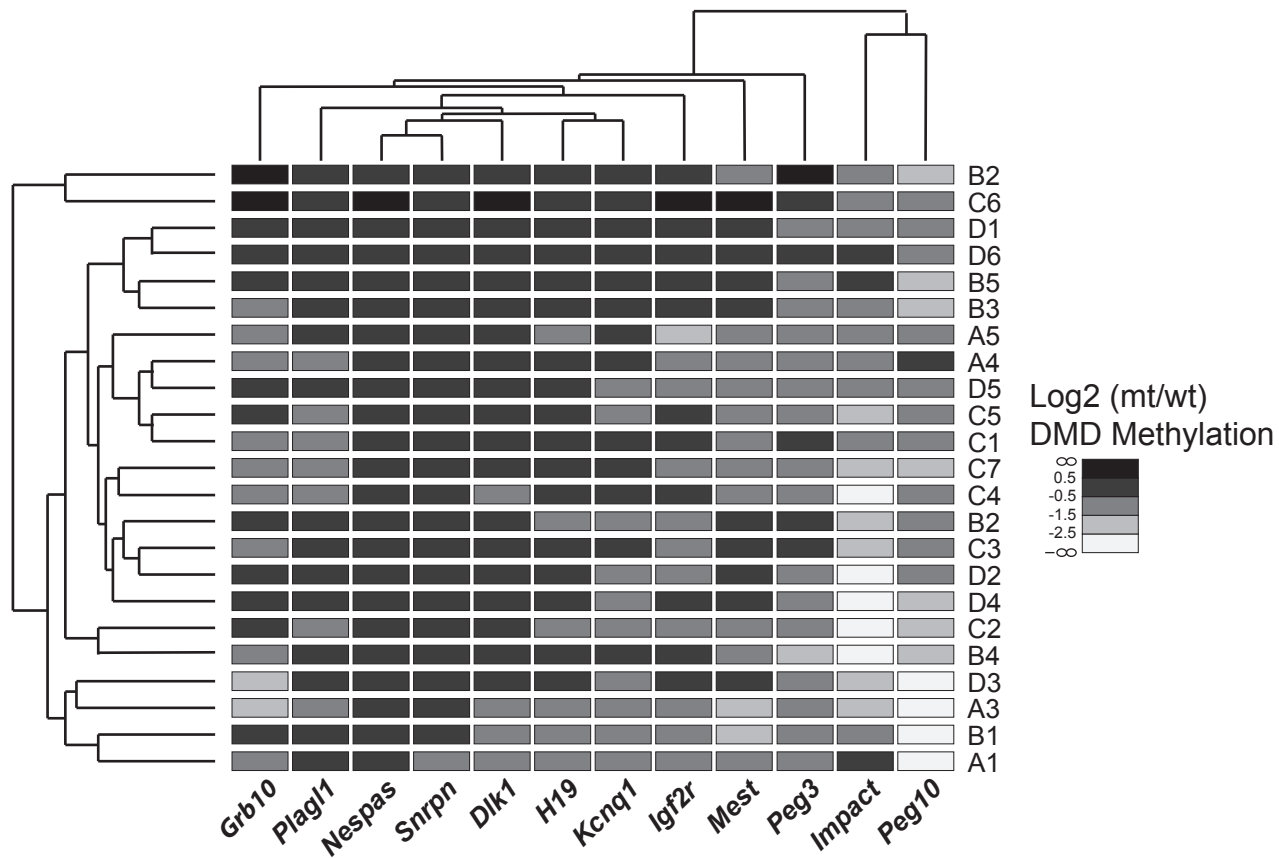

Supplement: S3 Fig — Data is shown as the log2 transformed ratio of mt:wt gDMD methylation. The heat map displays normally methylated gDMDs as dark boxes whereas loss of methylation is indicated by lighter shades. The upper and side dendrograms display linkage between imprinted gDMDs and DNMT1o-deficient samples respectively. Imprinted gDMDs are labeled across the bottom axis. DNMT1o-deficient samples are labeled down the right hand side by cohort litter (Letters A-D) and conceptus (Numbers 1–8). (PDF) [file pone.0135202.s003.pdf]

E17.5

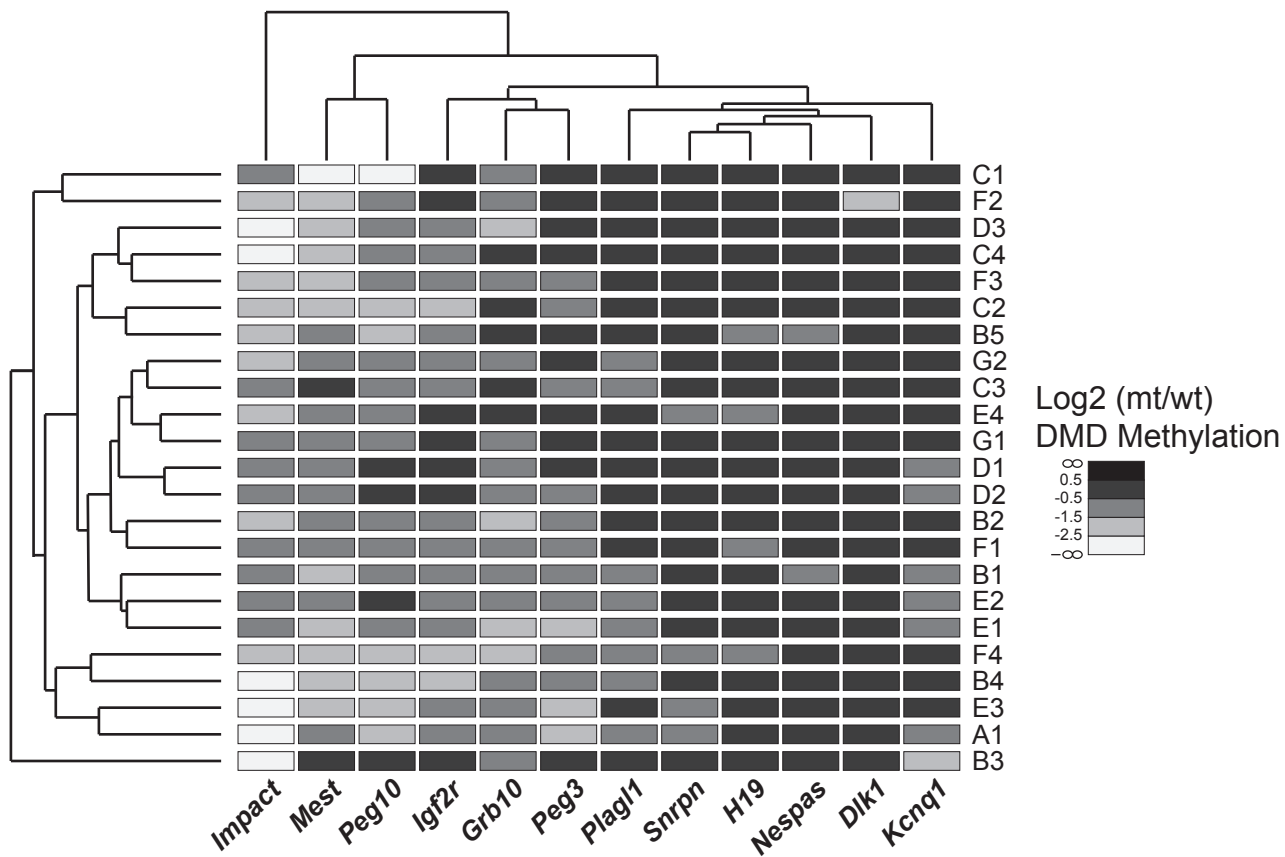

Supplement: S4 Fig — Data is shown as the log2 transformed ratio of mt:wt gDMD methylation. The heat map displays normally methylated gDMDs as dark boxes whereas loss of methylation is indicated by lighter shades. The upper and side dendrograms display linkage between imprinted gDMDs and DNMT1o-deficient samples respectively. Imprinted gDMDs are labeled across the bottom axis. DNMT1o-deficient samples are labeled down the right hand side by cohort litter (Letters A-G) and conceptus (Numbers 1–8). (PDF) [file pone.0135202.s004.pdf]

**A**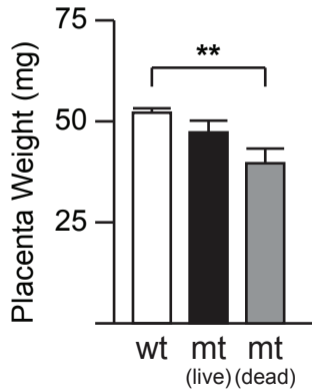**B**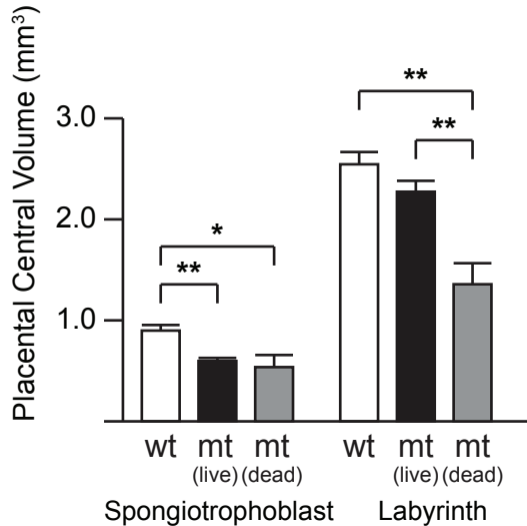**C**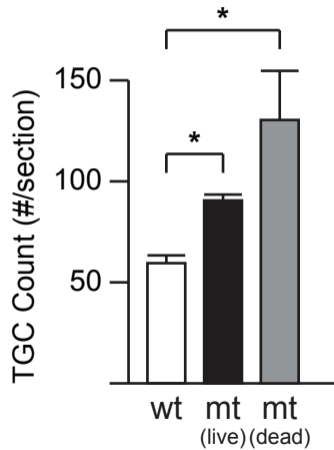

Supplement: S5 Fig — (A) Wet placenta weight, (B) Spongiotrophoblast and Labyrinth central volume, and (C) the number of TGCs per slide of wt, mt-live and mt-dead placental cohorts are displayed as white, black and gray bars respectively. Data are plotted as mean + SEM. *(P<0.05) and **(P<0.005) denote significant differences between wt, mt-live and mt-dead averages by the Rank-sum test. (PDF) [file pone.0135202.s005.pdf]

**A**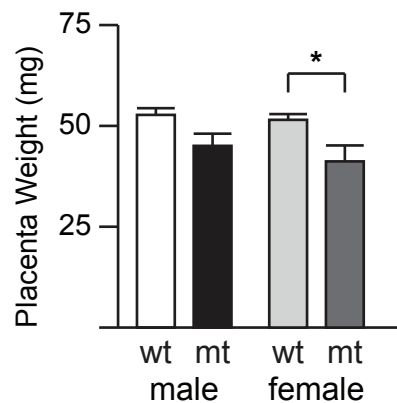**B**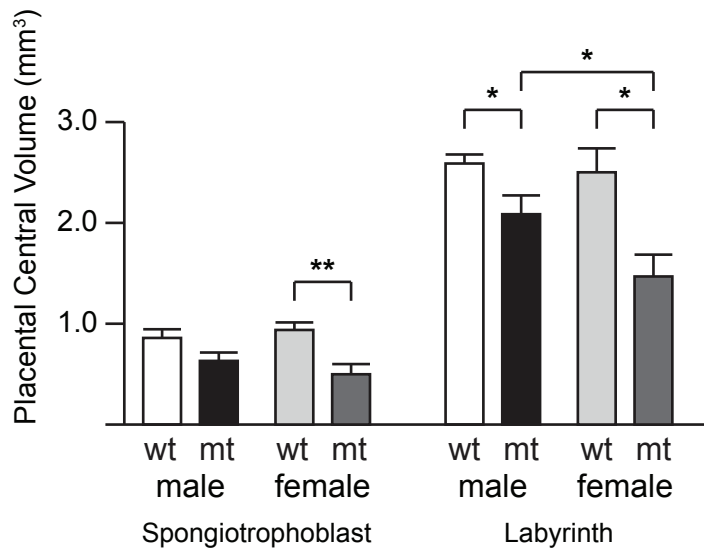**C**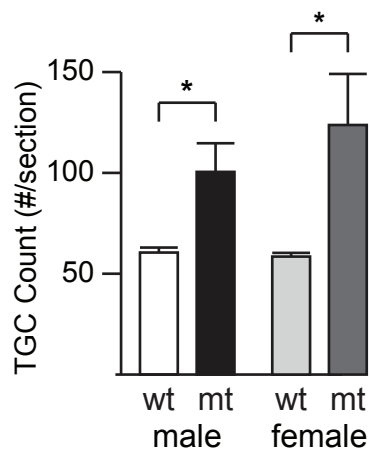

Supplement: S6 Fig — (A) Wet placenta weight, (B) Spongiotrophoblast and Labyrinth central volume, and (C) the number of TGCs per slide of wt-male, mt-male, wt-female and mt-female placental cohorts are displayed as white, black, light-gray and dark-gray bars respectively. Data are plotted as mean + SEM. *(P<0.05) and **(P<0.005) denotes significant differences between wt-male, wt-female, mt-male and mt-female averages by the Rank-sum test. (PDF) [file pone.0135202.s006.pdf]

**A**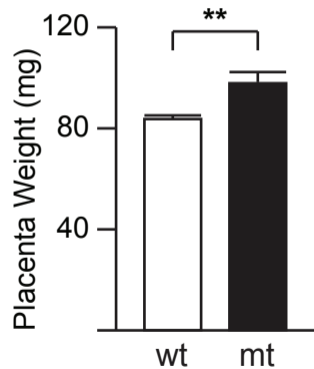**B**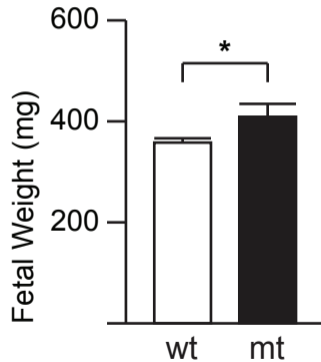**C**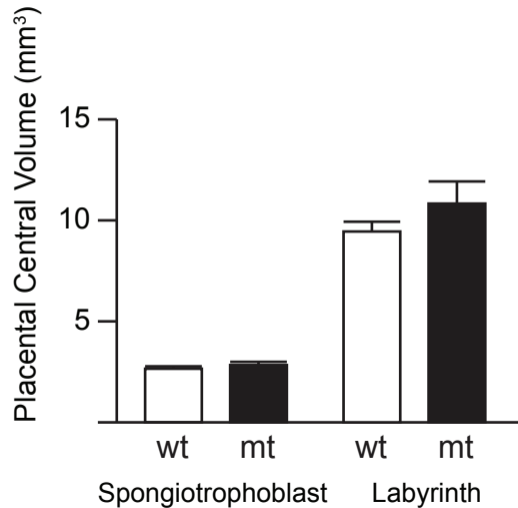

Supplement: S7 Fig — (A) Wet placenta weight, (B) Wet fetal weight, and (C) Spongiotrophoblast and Labyrinth central volume of wt and mt cohorts are displayed as open and filled bars respectively. Data are displayed as mean + SEM. * (P<0.05) and **(P<0.005) denote significant differences between wt and mt averages by the Rank-sum test. (PDF) [file pone.0135202.s007.pdf]

**A**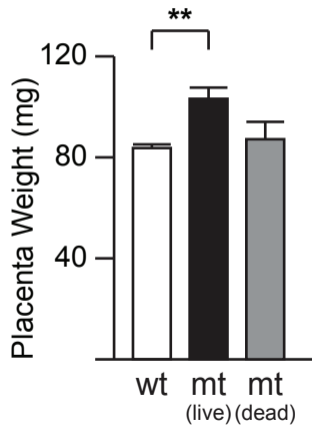**B**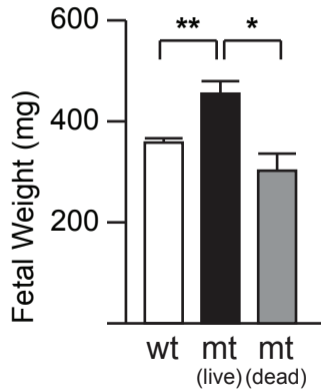**C**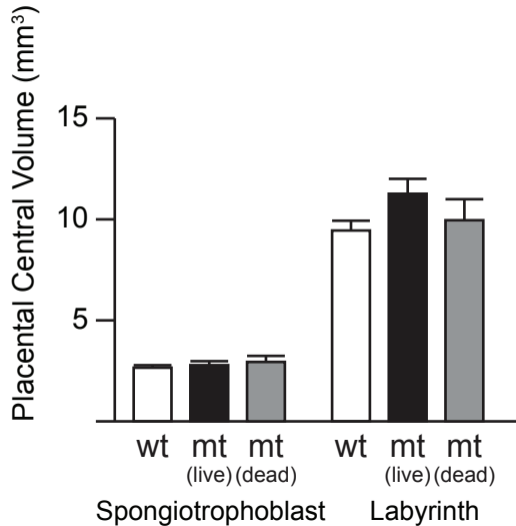

Supplement: S8 Fig — (A) Wet placenta weight, (B) Wet fetal weight, and (C) Spongiotrophoblast and Labyrinth central volume of wt, mt-live and mt-dead cohorts are displayed as white, black and gray bars respectively. Data are plotted as mean + SEM. *(P<0.05) and **(P<0.005) denote significant differences between wt, mt-live and mt-dead averages by the Rank-sum test. (PDF) [file pone.0135202.s008.pdf]

**A**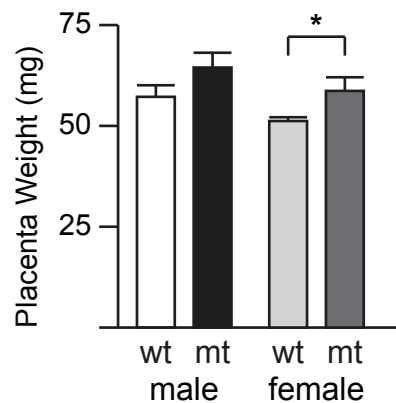**B**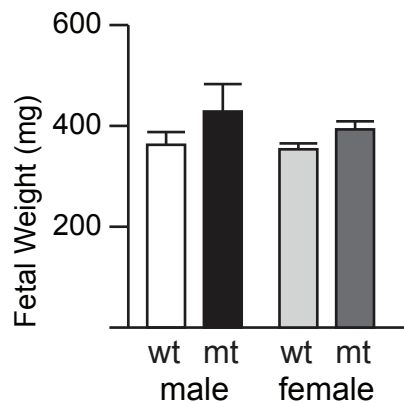**C**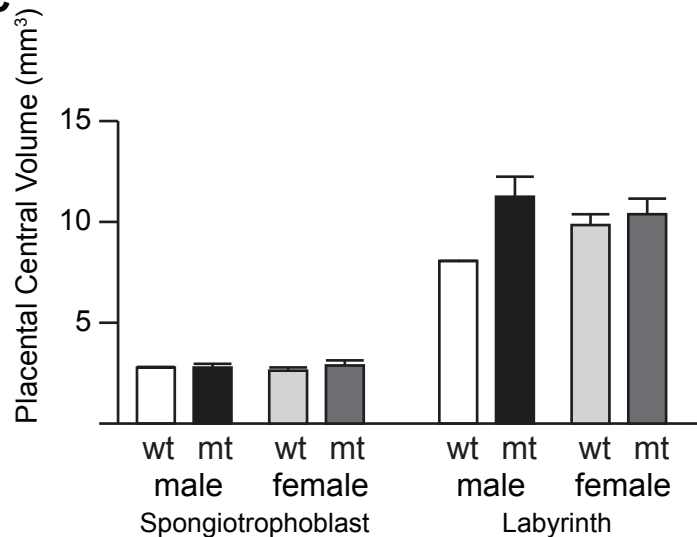

Supplement: S9 Fig — (A) Wet placenta weight, (B) Wet fetal weight, and (C) Spongiotrophoblast and Labyrinth central volume of wt-male, mt-male, wt-female and mt-female cohorts are displayed as white, black, light-gray and dark-gray bars respectively. Data are plotted as mean + SEM. *(P<0.05) and **(P<0.005) denotes significant differences between wt-male, wt-female, mt-male and mt-female averages by the Rank-sum test. (PDF) [file pone.0135202.s009.pdf]

**A**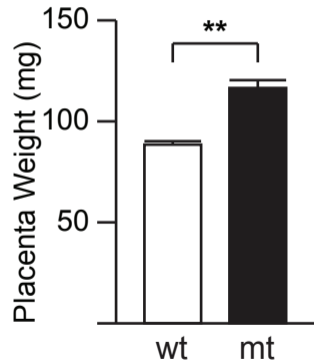**B**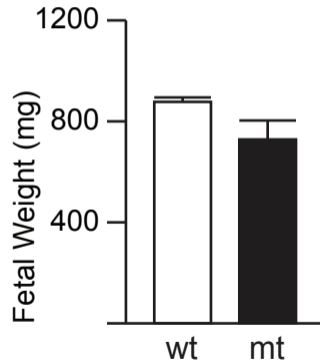

Supplement: S10 Fig — (A) Wet placental weights and (B) Wet fetal weights of wt and mt cohorts are shown as open and filled bars respectively. Data are displayed as mean +SEM. ** (P<0.001) by the Rank-Sum test. (PDF) [file pone.0135202.s010.pdf]

**A**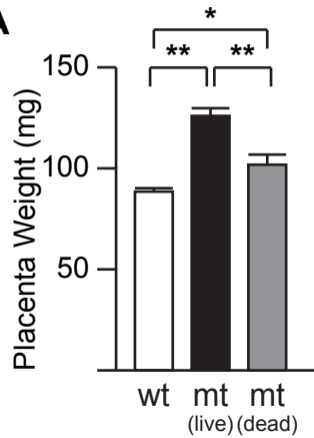**B**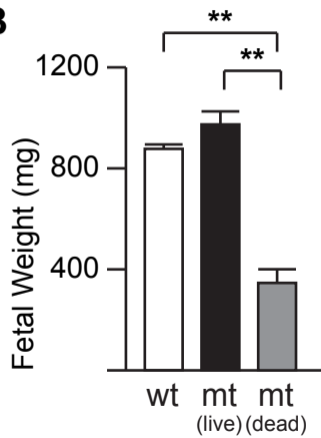

Supplement: S11 Fig — (A) Wet placenta weight and (B) Fetal weight of wt, mt-live and mt-dead conceptuses are displayed as white, black and gray bars respectively. Data are plotted as mean + SEM. *(P<0.05) and **(P<0.005) denote significant differences between wt, mt-live and mt-dead averages by the Rank-sum test. (PDF) [file pone.0135202.s011.pdf]

**A**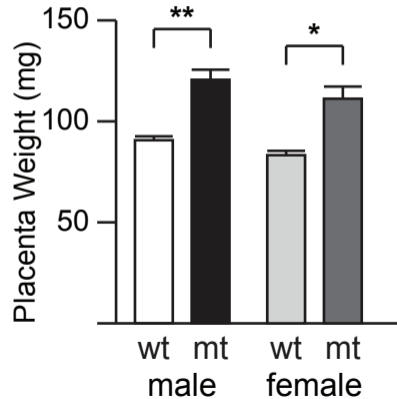**B**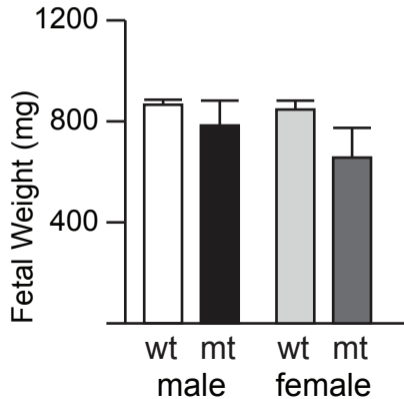

Supplement: S12 Fig — (A) Wet placenta weight, and (B) Wet fetal weight, of wt-male, mt-male, wt-female and mt-female conceptuses are displayed as white, black, light-gray and dark-gray bars respectively. Data are plotted as mean + SEM. *(P<0.05) and **(P<0.005) denote significant differences between wt-male, wt-female, mt-male and mt-female averages by the Rank-sum test. (PDF) [file pone.0135202.s012.pdf]

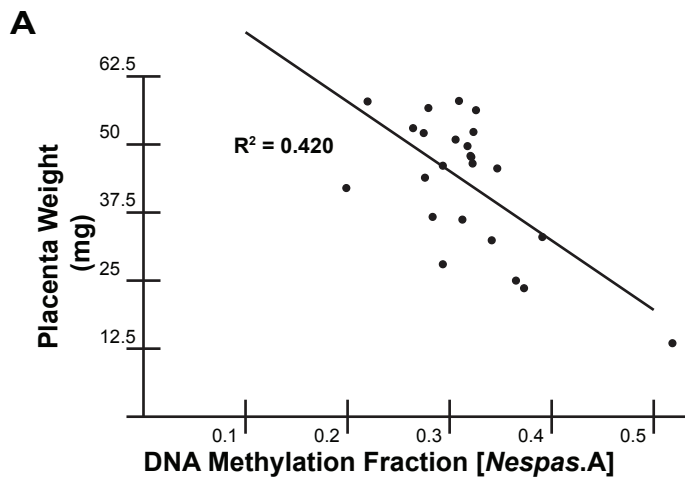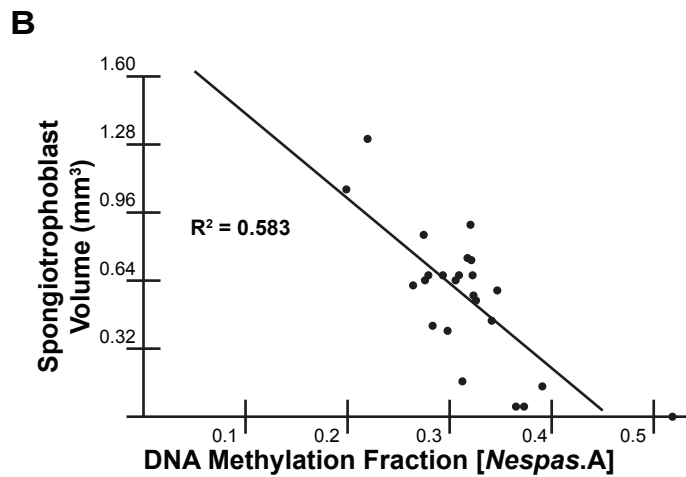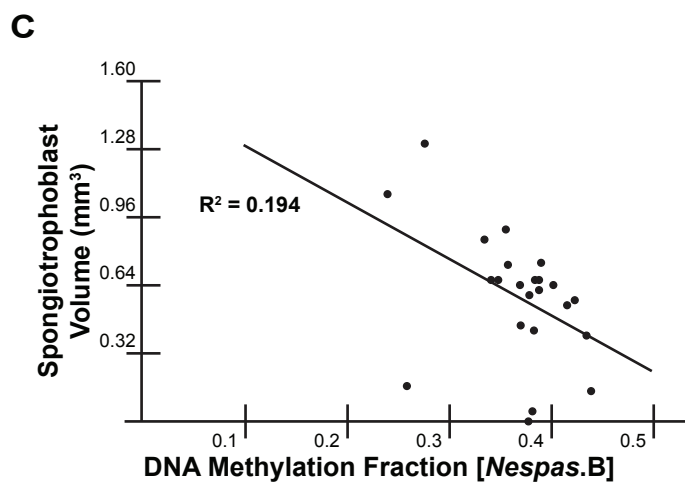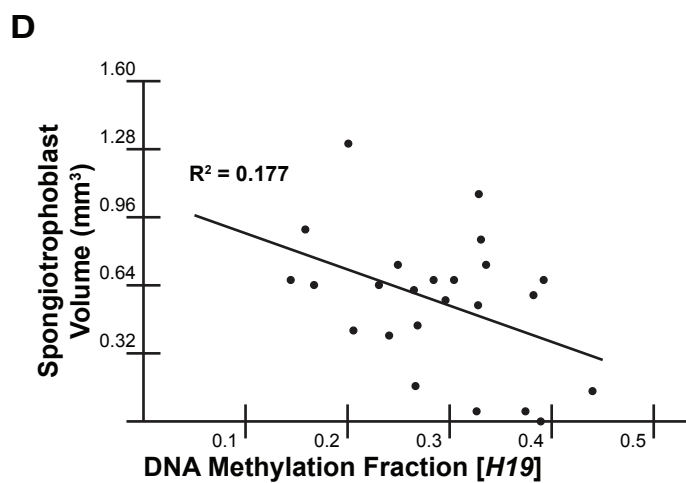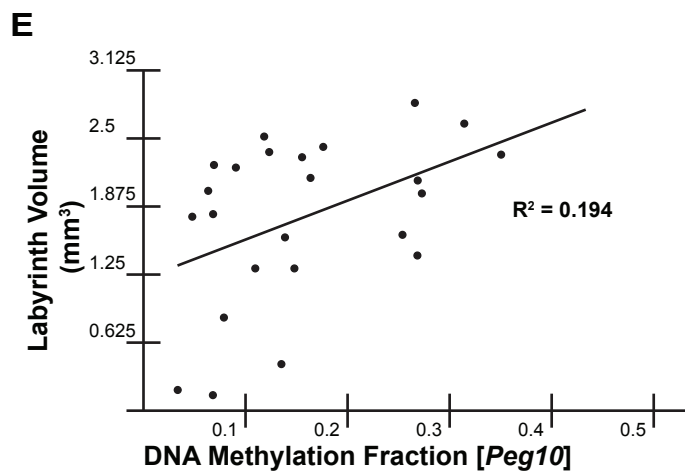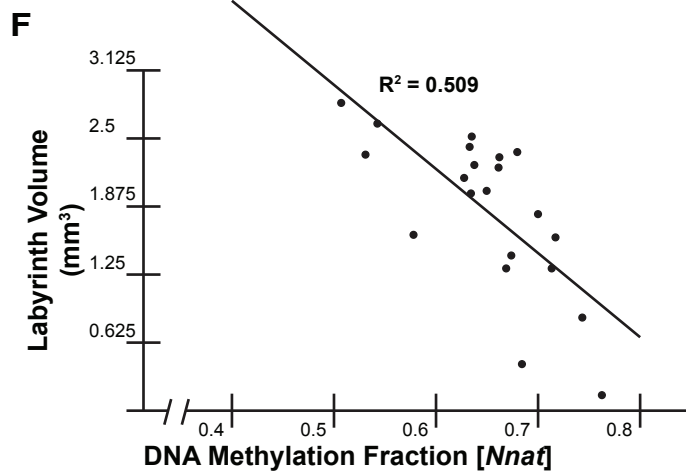

Supplement: S13 Fig — (A) Negative association between Nespas.A gDMD methylation and placental weight. (B) Negative association between Nespas.A gDMD methylation and spongiotrophoblast volume. (C) Negative association between Nespas.B gDMD methylation and spongiotrophoblast volume. (D) Negative association between H19 gDMD methylation and spongiotrophoblast volume. (E) Positive association between Peg10 gDMD methylation and labyrinth volume. (F) Negative association between Nnat gDMD methylation and labyrinth volume. R2 is unadjusted R-square value. (PDF) [file pone.0135202.s013.pdf]

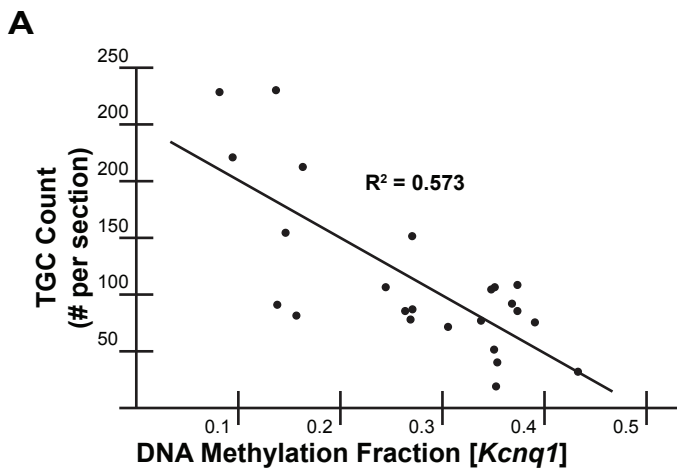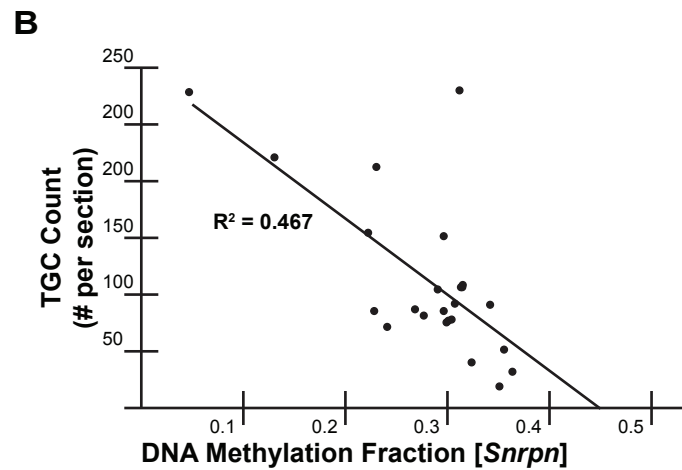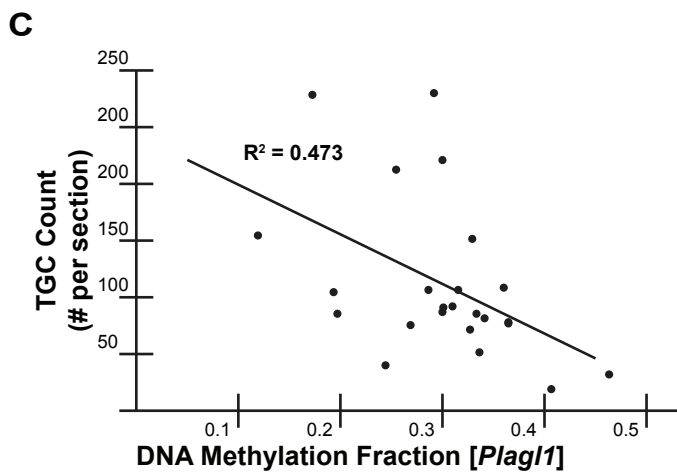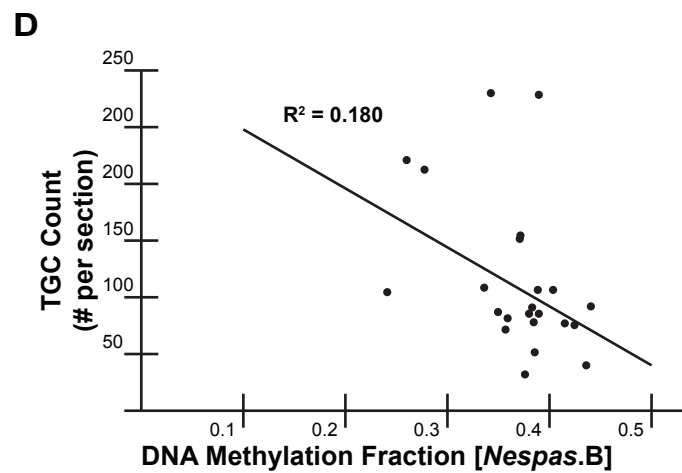

Supplement: S14 Fig — (A) Negative association between Kcnq1 gDMD methylation and TGC counts. (B) Negative association between Snrpn gDMD methylation and TGC counts. (C) Negative association between Plagl1 gDMD methylation and TGC counts. (D) Negative association between Nespas.B gDMD methylation and TGC counts. R2 is unadjusted R-square value. (PDF) [file pone.0135202.s014.pdf]
