# Supplementary material for: Partial Loss of Genomic Imprinting Reveals Important Roles for Kcnq1 and Peg10 Imprinted Domains in Placental Development
Source: PLoS One. 2015 Aug 4;10(8):e0135202. doi: 10.1371/journal.pone.0135202 (PMC4524636; doi:10.1371/journal.pone.0135202)
Supplement: S1 Table — Forward and Reverse sequence tags attached to each primer pair for EpiTYPER methylation analysis. Genomic coordinates of imprinted gDMDs from the most recent mouse genome build (GRCm38). Imprinted gDMD amplicons in bold were those used to calculate average imprinted gDMD methylation fraction in Fig 1A. (PDF) [file pone.0135202.s015.pdf]

EpiTYPER Bisulfite PCR Amplicon Primer Sequence and Coordinates

**5' Forward Tag**	AGGAAGAGAG
**3' Reverse T7 Tag**	CAGTAATACGACTCACTATAGGGAGAAGGCT

**Imprinted gDMD**		**Sequence (5' to 3')**	**Genomic Coordinates (GRCm38)**
***Dlk1*.A**	FWD	ATAGTATTGGTTTGGTATATATGGATG	12:109527424-109527853
	REV	CCATAACATAAACATAAAAATCCACAA	
Dlk1.B	FWD	GATGTGTTGTGGATTTAGGTTGTAG	12:109528138-109528138
	REV	ATCCCCTATACTCAAAACATTCTCC	
Grb10	FWD	AGGAGTTGTTTATTATTTGGATTATTGT	11:12025702-12026046
	REV	CTCTAAACTCCAAAACCCTTTTTCT	
H19	FWD	GTTGATGGTTTAGAATTTTATAAGTTAG	7:142581609-142581931
	REV	CACAATACCACTAAAAAAACAAAACA	
Igf2r	FWD	GATAGGAGGATTTAGAGGGTTTTGT	17:12742752-12743024
	REV	AACCCCATTATCTACAACTCAAACA	
***Impact*.A**	FWD	TTTGTATTAAGTAGGTTGTTTTAGGG	18:12972913-12973111
	REV	ACAACCAAACTAAAATTAACCAAACAA	
Impact.B	FWD	TTGTTTGGTTAATTTTAGTTTGGTT	18:12973084-12973497
	REV	TCATATAACAATACAACAAAACCTACTC	
Kcnq1	FWD	TGGAGAGTTTTTTTGTTTAGTTTGG	7:150481809 -150481430
	REV	CAAAACCACCCCTACTTCTATAAAC	
***Nespas*.A**	FWD	TGGGGGTTTTTGATTTTTTTATTTTG	2:174295281-174295599
	REV	TAAATCTCAACCACTAACCCACTCC	
Nespas.B	FWD	TTTTTTTTAGGGTTTTGTAGGTTAGATTTG	2:174295985-174296393
	REV	CCCCTCCTCCTTCTATTATAAACACC	
Mest	FWD	ATATGTTGGGGAGGGATTTTTTTAG	6:30737763-30738178
	REV	CAACAAAAACAACAAACAACAACTC	
Peg3	FWD	GATTTTGTTTGGGGGTTTTTAATATTGAT	7:6683342 -6683054
	REV	CCACCAACCCAAAATAAACATCTCT	
Plagl1	FWD	TATTTTTGTGGGGATGGAGGAATTA	10:13090467-13090791
	REV	ATCCCAACCCAAACTAAATAACAAA	
Peg10	FWD	TTAGGATTTGGTTATTGAAGGTTTG	6:4697732 -4697319
	REV	CCCCTCCTAAAATCTCTCTATATAAAAC	

EpiTYPER Bisulfite PCR Amplicon Primer Sequence and Coordinates

**5' Forward Tag**	AGGAAGAGAG
**3' Reverse T7 Tag**	CAGTAATACGACTCACTATAGGGAGAAGGCT

**Imprinted gDMD**		**Sequence (5' to 3')**	**Genomic Coordinates (GRCm38)**
Snrpn	FWD	TGTGATGTTTGTAATTATTTGGGAG	7:67150146 -67149901
	REV	CTAAAATCCACAAACCCAACTAACC	
Nnat	FWD	TTTAGGTGGTAAGAGGGTATTTAAGG	2:157560062-157560273
	REV	AATACATACTCACCTACAACAACAC	
Nap1l5	FWD	AGTTTGGAATTTTTTGTTAAATTTGG	6:58906694-58907060
	REV	CAACTACAAAACCTCTCTAAACCAAC	
Commd1	FWD	GGTAAGGTAGATAATTATTGTTTTAGTTGT	11:22972131-22972559
	REV	CATAAACCTACCCATACAATTACCC	
