# Supplementary material for: Partial Loss of Genomic Imprinting Reveals Important Roles for Kcnq1 and Peg10 Imprinted Domains in Placental Development
Source: PLoS One. 2015 Aug 4;10(8):e0135202. doi: 10.1371/journal.pone.0135202 (PMC4524636; doi:10.1371/journal.pone.0135202)
Supplement: S2 Table — N = 24, df = 21, model P-value = 1.54x10^-5^. (PDF) [file pone.0135202.s016.pdf]

Parameter	Estimate	SE	P-Value
Intercept	310	36.8	3.54×10^{-8}
Kcnq1 Regression Coeff (β)	-364	109	3.11×10^{-3}
Snrpn Regression Coeff (β)	-350	160	4.01×10^{-2}
