# Supplementary material for: Partial Loss of Genomic Imprinting Reveals Important Roles for Kcnq1 and Peg10 Imprinted Domains in Placental Development
Source: PLoS One. 2015 Aug 4;10(8):e0135202. doi: 10.1371/journal.pone.0135202 (PMC4524636; doi:10.1371/journal.pone.0135202)
Supplement: S3 Table — Only significant (P<0.05) associations are shown. Regression coefficient is either the logit (log odds ratio) for logistic regression for fetal viability, or the linear regression coefficient (β) for all other variables. (PDF) [file pone.0135202.s017.pdf]

Placental Phenotype	Imprinted gDMD	Regression Coefficient	Significance (P-value)
Fetal Viability	*Nespas*.B	-1.05	3.96×10^{-2}
Spongiotrophoblast Central Volume (mm^3)	*Impact*.B	-7.72	1.45×10^{-2}
	Mest	-4.49	1.87×10^{-2}
