# Supplementary material for: Partial Loss of Genomic Imprinting Reveals Important Roles for Kcnq1 and Peg10 Imprinted Domains in Placental Development
Source: PLoS One. 2015 Aug 4;10(8):e0135202. doi: 10.1371/journal.pone.0135202 (PMC4524636; doi:10.1371/journal.pone.0135202)
Supplement: S4 Table — Only associations with strong (P<0.075) are shown. The linear regression coefficient (β) is reported for both phenotypic variables. (PDF) [file pone.0135202.s018.pdf]

Placental Phenotype	Imprinted gDMD	Regression Coefficient	Significance (P-value)
Placental Weight (mg)	*Dlk1*.A	116	5.52×10^{-2}
Fetal Weight (mg)	*Igf2r*	2041	3.82×10^{-2}
	Mest	1668	7.07×10^{-2}
